# Patient and public engagement in priority setting: A systematic rapid review of the literature

**DOI:** 10.1371/journal.pone.0193579

**Published:** 2018-03-02

**Authors:** Elizabeth Manafò, Lisa Petermann, Virginia Vandall-Walker, Ping Mason-Lai

**Affiliations:** Patient Engagement Platform, Alberta SPOR SUPPORT Unit, Edomonton, Alberta, Canada; University of Exeter, UNITED KINGDOM

## Abstract

**Background:**

Current research suggests that while patients are becoming more engaged across the health delivery spectrum, this involvement occurs most often at the pre-preparation stage to identify ‘high-level’ priorities in health ecosystem priority setting, and at the preparation phase for health research.

**Objective:**

The purpose of this systematic rapid review of the literature is to describe the evidence that does exist in relation to patient and public engagement priority setting in both health ecosystem and health research.

**Data sources:**

HealthStar (via OVID); CINAHL; Proquest Databases; and Scholar’s Portal.

**Study eligibility criteria:**

i) published in English; ii) published within the timeframe of 2007—Current (10 years) unless the report/article was formative in synthesizing key considerations of patient engagement in health ecosystem and health research priority setting; iii) conducted in Canada, the US, Europe, UK, Australia/New Zealand, or Scandinavian countries.

**Study appraisal and synthesis:**

i) Is the research valid, sound, and applicable?; ii) what outcomes can we potentially expect if we implement the findings from this research?; iii) will the target population (i.e., health researchers and practitioners) be able to use this research?. A summary of findings from each of the respective processes was synthesized to highlight key information that would support decision-making for researchers when determining the best priority setting process to apply for their specific patient-oriented research.

**Results:**

Seventy articles from the UK, US, Canada, Netherlands and Australia were selected for review. Results were organized into two tiers of public and patient engagement in prioritization: Tier 1—Deliberative and Tier 2—Consultative. Highly structured patient and public engagement planning activities include the James Lind Alliance Priority Setting Partnerships (UK), Dialogue Method (Netherlands), Global Evidence Mapping (Australia), and the Deep Inclusion Method/CHoosing All Together (US).

**Limitations:**

The critical study limitations include challenges in comprehensively identifying the patient engagement literature for review, bias in article selection due to the identified scope, missed information due to a more limited use of exhaustive search strategies (e.g., in-depth hand searching), and the heterogeneity of reported study findings.

**Conclusion:**

The four public and patient engagement priority setting processes identified were successful in setting priorities that are inclusive and objectively based, specific to the priorities of stakeholders engaged in the process. The processes were robust, strategic and aimed to promote equity in patient voices. Key limitations identified a lack of evaluation data on the success and extent in which patients were engaged. Issues pertaining to feasibility of stakeholder engagement, coordination, communication and limited resources were also considered.

## Introduction

In Canada, the Strategy for Patient-Oriented Research (SPOR) was initiated to foster evidence-informed health care by bringing innovative approaches to the point of care for greater quality, accountability and accessibility [1]. The coalition of federal, provincial, and territorial partners is dedicated to integrating the patient voice into the research process to better ensure the patient voice and perspective is incorporated into policy and practice. Each SPOR SUPPORT (Support for People and Patient-Oriented Research and Trials) Unit offers locally accessible, multidisciplinary clusters of specialized research services, knowledge, and patient engagement to cultivate patient-oriented research and to facilitate a research culture change in response to the local needs and infrastructure gaps.

There is a critical opportunity to validate methodologies and frameworks for meaningful patient and public engagement in prioritization across the spectrum of research and decision-making activities. Given its relative infancy, there is a need to grow an evidentiary base about what works in achieving and sustaining productive patient engagement overall [2] and what does not [3]. This evidence will ultimately help to evaluate whether meaningful public and patient engagement priority setting impacts enhanced patient and family-centered care, service delivery, and health outcomes. There is a growing consensus identified in the literature that consulting with the public and patients is the necessary link between decision-makers and potential knowledge users [4, 5]. Indeed, without such engagement from the earliest stages, researchers and clinicians may ultimately miss the needs deemed as high priority by the end users [5].

Much of the current literature focusses on patient engagement across the health care delivery spectrum [6], although there is increased interest in implementing engagement opportunities in planning activities. Evidence suggests involvement in planning occurs most often at the ‘pre-preparation’ stage for identifying ‘high-level’ priorities in the health ecosystem (i.e., decision-making about health systems agendae and strategic planning opportunities) [7] and at the ‘preparation’ phase for research (i.e., identifying research topics, prioritizing topics, and developing/refining research questions) [8]. However, as evidenced by a recent scoping review conducted by this paper’s authors about patient engagement in health research, the literature is lacking in the provision of practical guidance on how to engage with the public and patients to obtain their perspectives. Based on these findings, it can also be inferred that a limited understanding of the best practices that focus on engaging patients and the public at the earlier stages of health ecosystem and health research priority setting also exists.

As such, members of the Patient Engagement Platform of the Alberta SPOR SUPPORT Unit (AbSPORU) conducted a rapid systematic review to describe the existing evidence about engaging patients and the public in the ‘pre-preparation’ and ‘preparation’ phases of health ecosystem and research priority setting. In this report the methods, findings, summary of key learnings, and suggested opportunities to support patient engagement in health planning and research are described. In particular, based on the findings, we will describe how patient engagement is used to promote collaboration and decision-making among patients, carers, researchers, health practitioners and decision-makers. The emphasis is on utilizing appropriate tools based on purpose, scope, and capacity particularly at the highest level of patient engagement, known as ‘deliberative engagement’ [9, 10].

## Definitions in public and patient engagement in prioritization

Similar to the findings of the previous scoping review led by the AbSPORU PE Platform [11] the language used to describe the spectrum of engagement across priority-setting activities is inconsistent. For example, Amba et al. [12] suggested that while ‘agenda setting’ and ‘priority setting’ are used interchangeably, they refer to different models and strategies to engage patients, carers, and clinicians in health research and beyond. Therefore, for the purpose of this review, the following definitions were operationalized and used in structuring the review:

### 1. Health ecosystem priority setting

Engaging the public and patients in priority setting in the health ecosystem was initially framed by principles of Participatory Action Research (PAR) which is defined by the engagement of public and patients in visioning or goal setting exercises [7]. Priority setting in the health ecosystem is framed by Khodyakov’s [13] definition of patients who are engaged in decision-making concerned with the planning and designing of programs. This implies patients being engaged in macro- or meso-level decisions more so than in clinical decision-making [14].

### 2. Research priority setting

Priority setting in health research is specifically guided by the Patient Outcome Centered Research Initiative’s (PCORI) definition of engaging patients in topic solicitation, prioritization, and framing of the research question [8, 15]. Specifically, this area of engagement focusses on what knowledge is (or questions are) valued most by patients and the public as they become experts in their health care experiences. They should have a say in determining research priorities and informing clinical decision-making [16].

At the same time, it is critical to note that despite the articulated definitions, the findings suggested an overlapping of *characteristics* for both priority setting activities i.e., health ecosystem and research, making a separate analysis of the results ineffective, limiting meaningful application to the end user. As such, the authors chose to organize the results using the distinguishing characteristics of engagement activities, which are the types and levels of engagement. These range from highly structured approaches with a high level of engagement to one-off consultations that are more limited in the opportunity for meaningful engagement. To operationalize the degree of public and patient engagement priority setting within the scope of this paper, Rowe and Frewer’s public engagement framework [17] was adapted to outline the three levels of public and patient engagement priority setting from least to most interactive [14]. The levels of engagement are integrated with Khodyakov’s [13] and Szelest’s [18] definitions of roles for patients in ‘health ecosystem priority setting’ and ‘health research priority setting’ respectively. This implies patients being engaged in decisions that inform direct clinical decision-making more so at the micro-level.

As noted by the International Association for Public Participation (IAP2) [19], engagement is broadly defined and can therefore encompass distinct levels of interactivity. The IAP2 Spectrum of Engagement (2014) clearly outlines five levels of engagement, which denote degrees of public participation. The spectrum suggests that the greater the degree of participation, the more influence the community has in its decision-making Vandall-Walker’s [20] Levels of Patient and Researcher Engagement in Health Research, adapted from the IAP2 spectrum, involves six levels of engagement, and further identifies the relative time, knowledge and monetary investment required by bothresearchers and patients, and mechanisms by which this can be addressed

Pratt et al. [21] assert that the deeper degrees of participation are better framed as ‘partnerships’. Indeed, deliberative processes of engagement extend beyond ‘focus group’ methods and instead add a range of opportunities for stakeholders to provide input [9]. This partnership level of engagement occurs when “the act of dialogue and negotiation serves to transform opinion in the members of both parties” [17].

**Fig 1:**
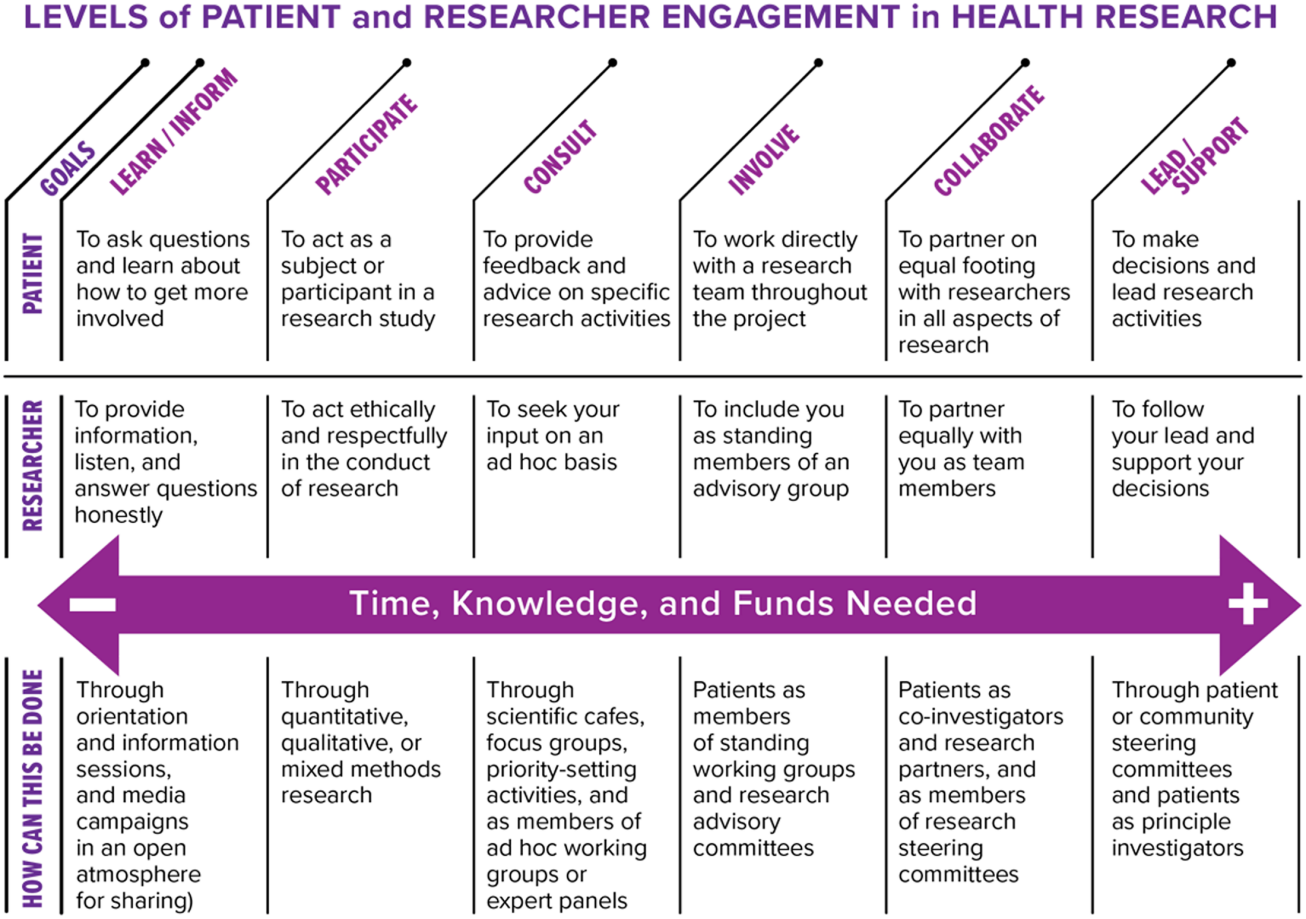
Levels of patient and researcher engagement in health research.

The public and patient engagement priority setting matrix developed for the specific purpose of this review is depicted in Table 1. The different levels of engagement are further categorized into ‘tiers’ of public and patient engagement priority setting. To note, as identified in the IAP2 Spectrum, patients and the public can also participate in a ‘one way’ communication process (i.e., Communicative), where they can simply learn about the priority setting processes. Examples include a public hearing or meeting, drop-in Centre or online information. This level of engagement is not included in Table 1 since the emphasis in this emergent area is to move away from one-way information transfer from decision-makers and researchers to the public, to a two-way process, as depicted by the Learn/Inform level of engagement noted by [20] (Fig 1). Furthermore, the role of the public and patient is to move beyond sharing of information amongst themselves and instead to seek out roles with researchers from the Consultation level to the Lead-level in priority-setting processes.

**Table 1:** Public and patient engagement matrix in health ecosystem & health research priority setting.

TIER	Levels of Engagement [10, 14]	Role of Public and Patients in Health Ecosystem Priority Setting [13]	Role of Public and Patients in Health Research Priority Setting [18]	Levels of Patient/Public and Researcher Participation [20]
**1** *Deliberative*	Dialogue and negotiation to transform opinions of both parties	Public and Patients are equal or lead stakeholders	Public and Patients collaborate by co-developing topics for research with researchers and other key groups	*Lead/Support*
**2** *Consultative*	Information is provided by the public to decision-makers, with limited interaction or formal dialogue	Public and Patients act as consultant and/or implementation advisors	Public and Patients consult about research topics and priorities that are most important to them	*Collaborate* *Involve* *Consult*

## Methods

A literature review was conducted using a systematic rapid review process, which is a more accelerated method of reviewing the literature compared with traditional systematic reviews. The PRISMA 2009 Checklist was used to endorse good reporting on the methods (S1 File). The advantages of this method are that it is quick, requires limited time and resources, and is functional in supporting informed decision-making. Emphasis is placed on locating and using synthesized research evidence and where not available, high-quality or recent primary studies [22]. A study protocol was first developed to describe the rationale and planned methods of the review (S2 File). This was prepared before the review and used when in its execution. A seven step process was used to undertake the review. All authors contributed and refined the review’s search strategy. Given its limited scope and timeline, the principal researcher (EM) conducted the literature search, applied the selection and critical appraisal criteria, selected the final article yield and extracted the relevant data. The established process provided the principal researcher (EM) with a framework to use in discussion with the other authors particularly when determining if an article met the selection or quality criteria.

### Step 1—Identify research questions

The following research questions were identified i) what are the methods of engaging the public and patients in ‘health ecosystem priority setting’ and ‘health research priority setting’ activities?; ii) What are the outcomes of engaging the public and patients in ‘health ecosystem priority setting’ and ‘health research priority setting’ activities?

### Step 2—Identify and select search terms

Next, given that this paper focusses on priority setting specifically, a review of key articles related to priority setting were identified and reviewed from the initial scoping review [11] to determine specific language that targetted the priority setting literature.

As well, to focus the search to health and health care literature, additional search terms were used to filter studies for inclusion that pertained to priority setting within the context of health and the health care system.

A combination of approaches was used to ensure that nuances in language were captured and documented as part of this research study. Search terms were adapted as needed to best meet the requirements of each database. Additional filters (e.g., geography, year of publication, language) were applied when available in the databases, to further refine the search and yield appropriate articles to the inclusion and exclusion criteria.

The search terms were as follows: patient engagement OR patient participation OR Involve AND research priority setting OR consensus build* OR research consult* AND health OR healthcare. (S1 Table)

### Step 3—Identify and select data sources

A three-pronged approach was used to collect data. First, specific and appropriate electronic databases were identified to explore the research questions with the assistance of Ryerson University Library Services accessible to the principle researcher (EM). Given the stated methods, the search strategy was narrowed to four relevant databases using all authors’ experiences in extracting relevant article yields and the principal researcher’s accessibility via the stated university’s library system: HealthStar (via OVID); CINAHL; Proquest Databases; and Scholar’s Portal.

Second, a targeted grey literature search was conducted. Each of the formal databases provided the option to also scan grey literature sources, which was a search included to broaden the reach of literature reviewed. Article titles that met the study selection criteria and clearly outlined the process, methods, and/or outcomes of priority setting, were inputted into the Ryerson University Library Services “Search Everything” database and PubMed’s “Single Citation Matcher”. Articles that were similar, relevant, or had been cited from the article title inputted, were reviewed for inclusion. Third, based on the identified process and methods of patient engagement, additional hand searching of the specific tools and/or frameworks was conducted (e.g., James Lind Alliance Priority Setting Partnership Manual).

The author feel confident in the search yield because of their combined use of various data sources as well as relying on literature from formal and manual search strategies.

### Step 4—Establish eligibility criteria

The following selection criteria were established based on the purpose and scope of the search. To be included in the review, articles had to address research priority setting at any or all points of the priority setting process and be: i) published in English; ii) published within the timeframe of 2007—Current (10 years) unless the report/article was formative in synthesizing key considerations of patient engagement in health ecosystem and health research priority setting; iii) conducted in Canada, the US, Europe, UK, Australia/New Zealand, or Scandinavian countries. Of those articles meeting the inclusion criteria, the following exclusion criteria were applied: does not clearly identify process, methods, and/or outcomes related to the health ecosystem or health research priority setting.

### Step 5—Apply critical appraisal criteria

To keep the research article yield focused within the predefined scope, the following quality selection criteria questions [23] were applied before selecting the article for data extraction: i) is the research valid, sound, and applicable?; ii) what outcomes can we potentially expect if we implement the findings from this research?; iii) will the target population (i.e., health researchers and practitioners) be able to use this research?. Articles that met all three of these criteria were selected for data extraction.

### Step 6—Search input and data extraction

Articles from this search were compiled into a comprehensive data extraction table. The data extracted included the resource citation, study methodology, study setting, technique, key activities, study outcomes, author considerations for barriers, and author consideration for facilitators,. From this information, the processes and activities described in the included articles were organized across the two aforementioned tiers of engagement i.e. Tier 1—Deliberative and Tier 2—Consultative. Few authors identified the level of participation explicitly using language such as ‘deliberative’ or ‘consultative’. As expected, most authors implicitly described levels of public and patient engagement. Therefore, articles were manually categorized based on the characteristics noted in the data extraction table.

### Step 7—Summarize key findings

A summary of findings from each of the respective processes was synthesized to highlight key information that would support decision-making for researchers when determining the best priority setting process to apply for their specific patient-oriented research. The data is reported in table format in the Findings to facilitate ease of comparison across priority setting processes.

## Results

Seventy articles were selected for this systematic rapid review (S1 Fig) (S2 Table). In Table 2, the characteristics of the selected articles are described.

**Table 2:** Public and patient engagement priority setting matrix of selected article characteristics.

Characteristic	Description (n)
Setting	UK = 29 US = 15 Canada = 13 Scandinavian countries = 9 Australia/New Zealand = 4
Primary focus	Health care improvement = 10 Mental health = 5 Cancer = 4 Spinal cord injury = 3 Kidney disease = 4 Diabetes = 2 Burns = 2 Chronic pain = 2 Disabilities = 2 Respiratory illness = 2 Skin conditions = 2
Primary Process and Activities	*Deliberative*: James Lind Alliance Priority Setting Partnership (UK) = 14 Dialogue Method (Netherlands) = 7 Global Evidence Mapping (Australia/New Zealand) = 2
	*Consultative*: Focus Groups/Workshops (e.g., Delphi) = 18 Key informant interviews/surveys = 9

### Overview of findings

‘Health ecosystem priority setting’ activities were most often identified at the ‘Consultative’ level of engagement (Tier 2). ‘Health research priority setting’ activities were most often identified at the ‘Deliberative’ (Tier 1) and ‘Consultative’ (Tier 2) engagement levels. In Table 3, examples of engagement processes and activities are organized according to the two identified levels of engagement.

**Table 3:** Tiered engagement processes and data collection activities for public and patient engagement prioritization.

Level of Engagement	1. ‘Health Ecosystem Priority Setting’ processes and activities	2. ‘Health research priority setting’ processes and activities
**TIER 1: Deliberative**	Citizens jury or consensus conference Negotiated rule making or task force Deliberate poll or planning cell	James Lind Alliance Priority Setting Partnership (UK) Dialogue Method (Netherlands) Deep Inclusion (US) CHoosing All Together (US) Global Evidence Mapping (Australia/New Zealand)
**TIER 2: Consultative**	Opinion poll or survey (Electronic or in-person)	Group meetings/Workshops Group/Individual surveys
	Referendum	n/a
	Consultation document with select persons or groups	Individual key informant interviews
	Focus groups	Focus groups
	Study circle or open space	n/a
	Standing citizens advisory panel	n/a

### Tier 1 public and patient engagement priority setting processes

A description of Tier 1 deliberative engagement activities in ‘health ecosystem priority setting’ were limited to Khodyakov’s [13] literature review, and were limited in description. The literature better emphasized highly-structured patient and public engagement planning processes and activities for research, including the James Lind Alliance Priority Setting Partnerships (UK) [10, 16, 24–35], the Dialogue Method (Netherlands) [25, 36–39], Global Evidence Mapping (Australia) [5], and the Deep Inclusion Method/CHoosing All Together (US) [21, 40]. While these research planning processes and activities differed, a common approach across Tier 1 public and patient engagement priority setting in research planning included gathering and analyzing identified research priorities by engaging patients and the public along with clinicians and researchers, followed by prioritization of topics through dialogue between all stakeholders. In Table 4, a summary of the Tier 1 public and patient engagement priority setting processes for health research is provided, including the aims, key characteristics, barriers, and facilitators of each of the identified models.

**Table 4:** Public and patient engagement priority setting process for health research summary.

TIER 1 Public and Patient Engagement Priority Setting Processes for Health Research				
	James Lind Alliance—Priority Setting Partnerships [10, 16, 24, 34, 35, 42, 43]	Dialogue Model	Global Evidence Mapping	Deep Inclusion / CHAT Method
	*Identify and prioritize public*, *patient*, *and clinician shared uncertainties about the effects of treatments across health conditions*	*Needs* and *priorities of patients and the public as a starting point for dialogue about research to improve health practice*	*Identify research questions which are mapped to available evidence for high-priority questions*	*Equity-oriented research priority setting by prioritizing input from minority or underserved populations*
**Key process/Steps**	Question gathering Question analysis Question prioritization Question integration Research Question or Treatment uncertainties summary	Question exploration Question consultation Question prioritization Question integration Question programming Question implementation Question Dissemination	Question development Question prioritization Evidence search and selection Data extraction Research Implementation	**A. Planning Phase** 1 Aims of priority setting process clarified 2 Priority setting mechanism identified 3 Ground rules established 4 Participation determined 5 Strategies to promote qualitative equality developed 6 Mode of non-elite participation determined **B. Identify Research Questions and Criteria Phase** 7 Health research topics/questions identified 8 Ranking criteria identified 9 Weights for ranking criteria identified **C. Selecting Priorities Phase** 10 Ranking criteria and weights to health research topics/question applied 11 Final set of priority health research topics/questions determined
**Sampling**	Users or ‘patients’ of a service Carers (e.g., care worker, relatives, spouses) Third sector representing organization Specialists (e.g., specialist knowledge on topic)	Patient/carer Researcher Decision-makers (including policy makers and researchers) Stakeholders are consulted *separately* to address potential asymmetry	Researchers Health professionals, Government agencies Patient support organizations People living with condition and a carer for someone with condition	Who—Number of participants in each category How—Strategies to address issues relating to disabilities, low socio-economic status, ethnic group representation When: Promoting entry points for engagement
**Cost**	*Rarely reported* Approximately $50,000CAD	*None reported*	*None reported*	*None reported*
**Timeline**	Up to 18 months	Up to 13 months	*Unclear* Preliminary literature review 5–8 weeks	*None reported*
**Outcomes**	Successful in setting priorities that are inclusive and objectively based Identifies differences in priorities of different stakeholders Presents opportunities to identify potential research gaps	Successful in prioritizing research questions specific to condition and populations	Successful in prioritizing research questions specific to condition Gaps in research are identified	Successful in prioritizing research focus groups specific to condition
**Strengths**	Robust, strategic multi-step approach Well recognized in literature for ability to identify priorities based on several treatment/condition ‘uncertainties’	Highly feasible	Uses a combination of activities to ensure prioritization of research questions is derived from multiple sources of evidence Identification of research gaps from multiple forms of evidence Synthesizes evidence in a meaningful way to capture priority esearch interest across diverse stakeholders	Clear process on ho to ensure equity in representation in priority setting

Overall, Tier 1 public and patient engagement priority setting research planning processes and activities demonstrated positive outcomes in setting priorities that were inclusive and objectively validated in the literature. Using the JLA process, for example, evidence of success was demonstrated by recent examples in acne treatments [33], prostate cancer research [34], wound care [10], kidney transplantation and dialysis [16, 32, 41], and dementia [31] among others. However, reporting of outcomes of the process was predominantly descriptive rather than evaluative. A mechanism for formal evaluation to measure the impact of these different planning processes on the quality of partnership and subsequent outputs is needed [10]. To note, the Dialogue Model process was tested over a 5-year period (2003–2007) for research priority setting based on seven case studies related to spinal cord injuries, neuromuscular diseases, renal failures, asthma/COPD, burns, diabetes, and intellectual disabilities [12]. While each case study demonstrated success in prioritizing research questions specific to the conditions and population, Amba et al. [12] noted that translating the Dialogue Model process to the particulars of the specific context was important, providing a useful opportunity to expand implementation and evaluation efforts of this process. This implies that ‘one size does not fit all’ in implementing public and patient engagement priority setting processes and activities

### Tier 2 public and patient engagement priority setting processes

Articles in which several different data collection activities were identified to engage patients were reviewed, such as through surveys and key informant interviews [44–51], focus groups [13, 14, 52–66], and patient panels [67]. While data collection activities differed, a common approach across Tier 2 included a two-step process. First, reviewing the literature and using expert opinion to determine a set of potential topics of interest; and second, using the topics determined as the basis for a survey for input by health professionals and the public [48]. Open-ended responses were critical [50]. Online surveys, key informant interviews, use of World Café or Dotmocracy facilitation tools provided the data [66].

Overall, evidence reviewed for Tier 2 public and patient engagement priority setting was more limited in describing comprehensive examples of facilitating patient engagement in health ecosystem and health research priority setting planning activities, although detailed techniques for structuring focus groups, surveys, and other group facilitation activities are available in the literature, but were deemed out-of-scope for this review. A lack of practical guidance on how to integrate public input with other forms of evidence, such as scientific articles, remains [14]. Reporting of operational details including cost, infrastructure, and timeline was limited or non-existent. Furthermore, there were few documented studies describing the effectiveness of the different data collection activities, as evaluation was typically informal and based on the perspectives of those organizing the engagement activities [14, 68].

Most often, success was not reliant on clear evaluation metrics but on the authors’ reviews of the process [56] or on opinions formed in reaching objectives that were not explicitly stated a priori [14, 56]. For example, Rideout et al. [52] identified that engaging patients at the macro level provided ample opportunity for shared research collaboration related to CVD and mental health. Working groups were formed with leadership provided by co-chairs: one from specific content areas and one from the public/community leaders. Conklin et al. [56] underscored that outcomes were inadequate due to insufficient periods of observation to measure the impact on policy and practice. However, authors were clear that local-level initiatives (vs. regional or national) were most likely to ultimately impact patient-centeredness and quality of care [45].

### Tier 1 and Tier 2 public and patient engagement priority setting limitations and opportunities

In Table 5, limitations and opportunities organized by individual, facilitation, and organizational perspectives are summarized [15, 37, 55, 59, 60].

**Table 5:** Barriers and enablers for public and patient engagement in prioritization in research.

	Barriers	Enablers
**Individual perspectives**	Common spoken language vs. medical language/ terminology Anticipated physician resistance to lay involvement Lack of content knowledge Group dynamics Tension among stakeholders Power/authority differential	Clear purpose for panel on what needs to be accomplished Presence of existing (informal) relationships Representation across different groups Public and Patient ownership of agenda Sense of urgency to address issues
**Facilitation perspectives**	Uncertainty of practicalities of promoting patient engagement Imprecise role of public and patient Insufficient time Omission of topics/in-exhaustive list Geographical limitations	Sufficient lead time Meetings held less frequently or with fewer stakeholders Skilled (trained) facilitator Shared topics ahead of time Mechanism to ensure patient voice is incorporated Stakeholder recruitment by networking with existing stakeholder groups expanded
**Organizational perspectives**	Professional attitudes towards public and patient engagement	Sufficient resources, infrastructure to support engagement opportunities (e.g., cost, time) Support from existing resources at institution

## Discussion

The key findings from this systematic rapid review of priority setting are supportive of the existing but limited recommendations outlined in the public and patient engagement priority setting literature [6, 10, 60, 63, 69, 70]. However, two predominant themes are worth discussing beyond the operational details of public and patient engagement priority setting activities, including the role of ethics in involving patients and the public as well as the opportunity for evaluation of engagement.

As referenced across the public and patient engagement priority setting and patient and public engagement literature, the risk of tokenism is high across all patient-engagement opportunities, limiting the output of the benefits of true engagement. In response, Pandya-Wood et al. [71] outlined the importance of developing an ‘ethically conscious’ framework for public and patient engagement priority setting. Addressing ethical considerations is critical for avoiding inadequate allocation of time that may result in a lesser impact on public engagement, tokenism, and potentially leading to public members being disenfranchised and unable to contribute fully to the study and as a result, disengaging. Ways to embed an ethical perspective in any engagement opportunity be it for patient and public engagement or public and patient engagement priority setting include: Timeframe: i) build realistic timeframes, including a research design stage which offers public involvement for at least two weeks to read, clarify, and provide feedback on the process; ii) Visibility: Make any public contributions visible; be transparent about how the public has been involved; iii) Accessibility: The public members involved in the research design stage require information, as do the rest of the research team, but communicated in way that they can access and understand, using plain language.

Finally, of critical importance to patient and public engagement in general and public and patient engagement priority setting in particular, is the role of evaluation in patient engagement in decision-making activities. Limited evaluative evidence may hinder future uptake of patient and public engagement in prioritization and decision-making exercises [10, 25, 43]. This includes the patient perspective about how, when, and why they are engaged and underscores the importance of continuing to catalogue public and patient engagement activities, including evaluation using validated tools to solicit the perspective of the public/patients, researchers, policy-makers, and organizations [58]. For example, Abelson et al. [58] reported on the development of the Public and Patient Engagement Evaluation Tool (PPEET) which includes three questionnaires designed to elicit feedback from: i) those who participate in Public and Patient engagement activities; ii) those who plan, execute, or sponsor Public and Patient engagement activities within an organization; and iii) those who provide leadership and capacity for Public and Patient engagement within their organizations. The questionnaires are guided by the key public and patient engagement principles of: Integrity of design and process; Influence and impact; Participatory culture; and Collaboration and Common Purpose. Chung et al. [44] also developed a publically available database for cataloguing current community engagement activities that can be leveraged for future engagement opportunities. This may also help with planning future steps to address engagement opportunities by supporting decision-makers with the tools needed to adequately, meaningfully, and effectively engage the public and patients, and in particular in public and patient engagement priority setting. With no consensus on the ‘gold standard’ of engagement [42], decision-makers require support for informed decision-making on effective public and patient engagement techniques. This may also help foster the much needed ‘buy in’ from patients, carers, researchers, and decision-makers.

## Limitations

The critical study limitations include challenges in comprehensively identifying the patient engagement literature for review, bias in article selection due to the identified scope, missed information due to a more limited use of exhaustive search strategies (e.g., in-depth hand searching), and the heterogeneity of reported study findings. These limitations can somewhat decrease the ability of the review to inform practical recommendations to support patient engagement in health research [72].

While the authors made the effort to focus the search for an article yield that was useful and meaningful, it is expected that not all articles that meet this review’s selection criteria were identified and selected. which is an inherent limitation of the stated methods. A lack of uniform reporting on indexing methods for an emerging area like patient engagement is a documented challenge in this emergent research area [73–74]. In itself, a wide range of terms are used across the patient engagement literature, to describe patient engagement in health research. This consideration was included in the design and execution of this systematic rapid review, although it is expected it may have impacted the yield.

In addition, the total number of articles selected for this review may have been limited by i) search terms that were not included as part of the search strategy; ii) limiting the number of databases used to conduct the search; and iii) limiting the grey literature search through databases instead of conducting this search manually (e.g., Google, Google Scholar).

Lastly, due to the heterogeneity of studies included, extracting specifically desired data from studies was a challenge, limiting the interpretation and generalizability of the results within the appropriate context of this review. With a longer timeline, additional resources, and a second researcher to conduct, review/appraise and select the relevant articles independently from the primary researcher the study’s yield may have been further strengthened. Additional research that emphasizes useful and transparent findings of patient engagement using the GRIPP2 (Guidance for Reporting Involvement of Patients and Public) [75] checklist criteria for patient and public involvement may better ensure that this research focus continues to evolve in a manner that is more meaningful to patients and health outcomes.

At the same time, these limitations are mitigated by: i) inclusion of articles from AbSPORU PE Platform-led scoping review of patient engagement in health research [11]; ii) Inclusion of articles of relevance sent by staff from the Alberta SPOR Support Unit; iii) review of reference lists and input of single citation matchers for identified articles; iv) widening of the typical timeframe for selected articles (i.e., 3–5 years) given the emergent nature of the patient engagement enterprise.

## Recommendations

Additional opportunities to leverage the existing recommendations are offered for consideration. The authors suggest the following to focus efforts on cataloguing public and patient engagement priority setting initiatives:

1. Maintain adequate data monitoring, collection, and evaluation to support continued interest in public and patient engagement priority setting and to encourage ‘buy in’ from public/patients, researchers, and decision-makers;
2. Describe operational details of public and patient engagement priority setting ‘best practices’: There is need for better reporting of operational details of public and patient engagement priority setting that both worked and did not work, including process activities, sampling, cost, time, outputs and evaluation. This will help inform decision-making on which processes and activities will best suit the given circumstance or context for public and patient engagement priority setting activities. An example of one reporting tool includes creating and managing a data collection template for ongoing public and patient engagement priority setting activities;
3. Prioritize evaluation of public and patient engagement priority setting initiatives: To supplement existing descriptive outcomes, evaluative outcomes of the impact of patient and public engagement in health ecosystem and health research priority setting are needed to build a robust evidence-base for patient and public engagement specifically in priority setting activities. Priority setting activities are a useful entry for patient and public engagement activities across the research spectrum; and
4. Integrate knowledge mobilization opportunities: While evaluation of findings is important, it is equally important to ensure a knowledge mobilization plan is integrated into the public and patient engagement priority setting process to ensure patient and public engagement priorities are translated into action.

It is intended that the AbSPORU PE Platform will consider and take action on these points, as well as on the evidence synthesized in this review, to support future decision-making with research teams about engaging in Public and Patient Engagement activities in health research priority setting.

## Supporting information

S1 File Study PRISMA checklist. (DOC)

S2 File Study protocol. (DOCX)

S1 Table Study search output. (DOCX)

S2 Table Study data extraction table. (DOCX)

S1 Fig Study PRISMA flow diagram. (DOC)
