# Supplementary material for: Patient and public engagement in priority setting: A systematic rapid review of the literature
Source: PLoS One. 2018 Mar 2;13(3):e0193579. doi: 10.1371/journal.pone.0193579 (PMC5834195; doi:10.1371/journal.pone.0193579)
Supplement: S2 File — (DOCX) [file pone.0193579.s002.docx]

1. **Study Rationale:**

The purpose of this systematic rapid review of the literature is to describe the evidence that does exist in relation to patient and public engagement priority setting in both health ecosystem and health research.

**2. Research Questions**

1. What are the methods of engaging patients in priority setting research activities?
2. What are the outcomes of engaging patients in priority setting in health research process and patient outcome?

**3. Scope:**

- ‘Patient’ is used as an overarching term to include individuals with personal experience of a health issue and informal caregivers, including family and friends
- ‘Priority setting research activities’ is framed by Izabela Szelest’s *Role for Patients in Research* (n.d.):
  1. Through community representation and organizations help inform research priorities
  2. Be consulted about research topics and priorities important to them
  3. Collaborate and co-develop with researchers and other key groups topics for research

**4.** **Search Strategy:**

- *Primary search strategy:*
  - Formal literature review (Outlined in 4. Conducting Search)
- *Secondary search strategy:*
  - Inclusion of articles from patient engagement in health research scoping review (data extraction table)
  - Inclusion of article sent by staff from Alberta SPOR Support Unit
  - Review reference list and input single citation matcher for identified articles in PubMed

**5. Conducting Search**

*Search terms:*

| Patient engagement OR patient participation OR patient consultation OR patient involvement | **AND** | Research priority setting OR consensus build* OR research consult* | **AND** | Health OR health care |
| --- | --- | --- | --- | --- |

*Inclusion criteria*

- Peer-reviewed journal articles, research reports, guidelines documents
- Published in English or French
- Published within the timeframe of 2006 - Current (10 years)
- Studies conducted in Canada, US, Europe, UK, Australia and New Zealand

*Exclusion criteria*

- Patient Education: Patient-engagement in client care (e.g., Patient Portals, eMRs) to support decision-making/improvements in quality of care/uptake of care/intervention.

**6. Data sources**

*Research Indices*

- Healthstar (via OVID)
- CINAHL
- Proquest Databases
- Scholar’s Portal

**7. Critical appraisal criteria:**

Rychetnik et al^[[1]](#footnote-1)^ suggest the following when selecting research articles:

1)     Is the research valid, sound and applicable?

2)     What outcomes can I expect if I implement this research?

3)     Will my target population be able to use this research?

**8. Data extraction variables**

- Source/Citation
- Year
- Study Methodology
- Study setting
- Condition/Context
- Method of engagement in priority setting (community representation, consultation, collaboration)
- Outcomes/Impact on research
- Key author findings
- Data extractor comments

1. Rychetnik L, Frommer M, Hawe P et al. Criteria for evaluating evidence on public health interventions. *J Epidemiol Community Health* 2002; 56: 119–27. [↑](#footnote-ref-1)
