# Supplementary material for: Patient and public engagement in priority setting: A systematic rapid review of the literature
Source: PLoS One. 2018 Mar 2;13(3):e0193579. doi: 10.1371/journal.pone.0193579 (PMC5834195; doi:10.1371/journal.pone.0193579)
Supplement: S1 Table — (DOCX) [file pone.0193579.s003.docx]

**A. Priority Setting: Formal Database Search Output**

*Search conducted April 26, 2017*

*Used HealthStar (OVID), CINAHL, Scholar’s Portal and Proquest*

**Table A1. Search strategy used for HealthStar Database (2007 – April 2017)**

| **#** | **Search Term & Limits** | **Number of Retrievals** | **Initial Screen** |
| --- | --- | --- | --- |
| 1 | Patient engagement OR participat* Or Involv* AND priority setting OR consensus build* OR research consult* AND health OR healthcare*mp | 94 | 31 |

**Table A2. Search strategy used for CINAHL Database (2007 – April 2017)**

| **#** | **Search Term & Limits** | **Number of Retrievals** | **Initial Screen** |
| --- | --- | --- | --- |
| 1 | Patient engagement OR participat* Or Involv* AND priority setting OR consensus build* OR research consult* AND health OR healthcare* | 1225 |  |
| 4 | Limit to 2007-Current, English and French, Humans and Peer Reviewed | 154 | 11 |

**Table A3. Search strategy used for Scholars Portal Database (2007 – April 2017)**

| **#** | **Search Term & Limits** | **Number of Retrievals** | **Initial Screen** |
| --- | --- | --- | --- |
| 1 | Patient engagement OR participat* Or Involv* AND priority setting OR consensus build* OR research consult* AND health OR healthcare*mp | 22378 |  |
| 4 | Limit to 2007-Current, English and French, Humans and Peer Reviewed | 22 | 5 |

**Table A4. Search strategy used for ProQuest (2007 – April 2017)**

| **#** | **Search Term & Limits** | **Number of Retrievals** | **Initial Screen** |
| --- | --- | --- | --- |
| 1 | Patient engagement OR participat* Or Involv* AND priority setting OR consensus build* OR research consult* AND health OR healthcare*mp | 77037 |  |
| 4 | Limit to 2007-Current, English and French, Humans and Peer Reviewed | 59 | 9 |
