# Supplementary material for: Patient and public engagement in priority setting: A systematic rapid review of the literature
Source: PLoS One. 2018 Mar 2;13(3):e0193579. doi: 10.1371/journal.pone.0193579 (PMC5834195; doi:10.1371/journal.pone.0193579)
Supplement: S2 Table — (DOCX) [file pone.0193579.s004.docx]

| **Source Citation** | **Study**  **Description** | **Technique, Description and Outcome (when available)** | **Author considerations of barriers and study limitations** | **Author considerations for facilitation and future research** |
| --- | --- | --- | --- | --- |
| Abelson, Julia, Kathy Li, Geoff Wilson, Kristin Shields, Colleen Schneider, and Sarah Boesveld. "Supporting Quality Public and Patient Engagement in Health System Organizations: Development and Usability Testing of the Public and Patient Engagement Evaluation Tool." Health Expectations 19.4 (2016): 817-27. | *Design:* Experimental  *Setting:*  Canada  *Technique:*  Focus Group/ Group Meeting/ Workshop | The evaluation tool was developed through an iterative, collaborative process informed by a review of published and grey literature and with the input of Canadian PPE researchers and practitioners. Over a 3-year period, structured e-mail, telephone and face-to-face exchanges, including a modified Delphi process, were used to produce an evaluation tool that includes core principles of  high-quality engagement, expected outcomes for each principle and three unique evaluation questionnaires that were tested and revised with input from 65 end users. | None | This study describes a novel approach of collaboration of researchers, practitioners in the co-design of a comprehensive PPE evaluation tool aimed at three distinct respondent groups for use in a wide range of health system organization settings |
| Abma, Tineke A., and Jacqueline E. W. Broerse. "Patient Participation as Dialogue: Setting Research Agendas." Health Expectations : An International Journal of Public Participation in Health Care and Health Policy 13.2 (2010): 160. | Design: Non-experimental  Setting: Scandinavia  Technique: Dialogue Model | The Dialogue Model is grounded in participatory and interactive approaches and has been adjusted on the basis of pilot work. It has six phases: exploration; consultation; prioritization; integration; programming; and implementation. These phases are discussed and illustrated with a case description of research agenda setting relating to burns. | The Dialogue Model uses a convenience sample, which is fine as long as the research is qualitative and explorative. However, since the status of the outcome is to  increase the legitimacy of the research agenda, some may argue that the issue of a representative sample becomes highly relevant. | Consider the extent in which research priorities of patients are representative of entire patient population.  Determining interest of researchers and other professionals in conducting this technique  What structural changes are required to further facilitate adoption of this method |
| Abma, Tineke A. "Patients as Partners in a Health Research Agenda Setting: The Feasibility of a Participatory Methodology." Evaluation & the Health Professions 29.4 (2006): 424-39. | *Design:* Case study/ series  *Setting:*  Scandinavia  *Technique:*  Focus Group/ Group Meeting/ Workshop | Focus group/key informant sessions used to prioritize health care priorities | Limited ability to develop mutual trust and commitment  Inclusion fo patients surrounding with concerns  Concerns on how to foster meaningful patient participation | Consideration of ‘who’ should be involved in study to ensure methodology reflects diversity |
| Arkind, Jill, Sonja Likumahuwa-Ackman, Nate Warren, Kay Dickerson, Lynn Robbins, Kathy Norman, and Jennifer E. Devoe. "Lessons Learned from Developing a Patient Engagement Panel: An OCHIN Report." Journal of the American Board of Family Medicine : JABFM 28.5 (2015): 632. | *Design:*  Non-experimental  *Setting:* USA  *Technique:*  Patient Engagement Panel | PEP demonstrates the feasibility of incorporating a patient voice throughout the research life cycle, with opportunities to amplify it in additional  non-research initiatives. A multistep, collaborative process was successfully used to identify and recruit patients, develop a shared vision, and agree  on equitable compensation models.  lessons learned through this work are transferrable  to quality improvement and innovations in health  care settings that would benefit from patient involvement | Challenge in recruiting PEP members representative of a range of different backgrounds and ‘hard-to-reach groups’, including homeless, single parent, less-educated, disabled and patients living in poverty  How to create a sustainable funding structure | How do researchers and patients in PEP equally value through participation |
| Armstrong, Melissa J., C. Daniel Mullins, Gary S. Gronseth, and Anna R. Gagliardi. "Recommendations for Patient Engagement in Guideline Development Panels: A Qualitative Focus Group Study of Guideline-naïve Patients." PLoS One 12.3 (2017): n. pag. | *Design:*  Non-experimental  *Setting:*  USA  *Technique:*  Focus Group/ Group Meeting/ Workshop | Focus group/key informant sessions used to prioritize health care priorities | Main barriers include:   Spoken language; Medical terminology; anticipated physician resistance to lay involvement; lack of content knowledge; Group dynamics and insufficient time | Consider panel composition, the number of patients to physicians and ensuring multiple patient representativeness as ideal  Patient panelists should be knowledgeable and unbiased and have relevant expertise and be willing to actively contribute and ask questions  Look for good dynamic relationship between patient and physician  Establish purpose of panel and what needs to e accomplished; Share the topic ahead of time, use a skilled facilitator and have a planned mechanism to ensure patient voice is incorporated |
| Batchelor, J. M., M. J. Ridd, T. Clarke, A. Ahmed, M. Cox, S. Crowe, M. Howard, S. Lawton, M. Mcphee, A. Rani, J. C. Ravenscroft, A. Roberts, and K. S. Thomas. "The Eczema Priority Setting Partnership: A Collaboration between Patients, Carers, Clinicians and Researchers to Identify and Prioritize Important Research Questions for the Treatment of Eczema." British Journal of Dermatology 168.3 (2013): 577-82. | *Design:*  Non-experimental  *Setting:*  UK  *Technique:*  JLA/PSP | Step 1. Initiation (Identification of potential partner organizations and individuals ensuring representation of patients, carers and health care professionals  Step 2. Consultation. Participants invited to submit up to five eczema treatment uncertainties.  Step 3. Collation. Any non-questions or uncertainties not related to eczema or its treatment removed. Unique questions combined into 'indicative uncertainties' and reworded into standard question format  Stage 4. Ranking. Participants asked to select top 10 indicative uncertainties  Stage 5: Workshop. Workshop to formulate research questions based on shared (patient/carer and healthcare professionals) uncertainties | Concerns on tokenism  Concerns on equity in patient voice with physicians | None |
| Bodison, Stefanie C., Ibrahima Sankaré, Henry Anaya, Juanita Booker‐Vaughns, Aria Miller, Pluscedia Williams, Keith Norris, and Community Engagement Workgroup. "Engaging the Community in the Dissemination, Implementation, and Improvement of Health‐Related Research." Clinical and Translational Science 8.6 (2015): 814-19. Web. 10 May 2017. | *Design:*  Non-experimental  *Setting:*  USA  *Technique:*  n/a | This article summarizes the key challenges relating to engaging patients in priority setting activities and opportunities for consideration to address the barriers | Barrier 1: Historically, communities of interest have had little influence over the “problems” to be studied in health-related research  Barrier 2: Often, during the development of research projects, key stakeholders are left out of the process  Barrier 3: Cultural differences and health disparities often limit community engagement  Barrier 4: Dissemination of research findings seldom reach and/or are meaningful to the communities of study | Potential Solution 1: Adopt a clear, consistent framework of CPPR that engages the community in all phases of the research pipeline  Potential Solution 2: Develop a transdisciplinary approach to solving community-based “problems”  Potential Solution 3: Potential Solution: Acknowledge that health disparities and cultural differences exist, and put supports in place to minimize the effect of said differences  Potential Solution 4: Commit to communicating research findings via multiple methods |
| Boivin, A., P. Lehoux, R. Lacombe, J. Burgers, and R. P. Grol. "Involving Patients in Setting Priorities for Healthcare Improvement: A Cluster Randomized Trial." Implementation Science 9 (2014): 24. | *Design:*  Experimental  *Setting:*  Canada  *Technique:* Survey and key informant interviews | Randomized trial. Communities in a Canadian region were required to set priorities for improving chronic disease management in primary care, from a list of 37 validated quality indicators. Patients were consulted in writing, before participating in face-to-face deliberation with professionals (intervention). Professionals established priorities among themselves, without patient involvement (control). The primary outcome was the level of agreement between patients’ and professionals’ priorities. Secondary outcomes included professionals’ intention to use the selected quality indicators, and the costs of patient involvement. | Average cost of public involvement was $9427 per site. Patient involvement increased the cost of the prioritization process by 17% compared with priority-setting by professionals alone. Most of the patient involvement costs were incurred by compensation of participants' time, meal and travel expenses, coordination of patient recruitment and hiring of a professional faciltiator.  Time to agree on common priorities was on average 10% longer in intervention site | None |
| Boney, Oliver, Madeline Bell, Natalie Bell, Ann Conquest, Marion Cumbers, Sharon Drake, Mike Galsworthy, Jacqui Gath, Michael P. W. Grocott, Emma Harris, Simon Howell, Anthony Ingold, Michael H. Nathanson, Thomas Pinkney, and Leanne Metcalf. "Identifying Research Priorities in Anaesthesia and Perioperative Care: Final Report of the Joint National Institute of Academic Anaesthesia/James Lind Alliance Research Priority Setting Partnership." BMJ Open 5.12 (2015): E010006. | *Design:*  Non-experiential  *Setting:*  UK  *Technique:*  JLA-PSP | Six hundred and twenty-three individuals, almost all (98%) aged between 25 and 75 years old, submitted  1420 research suggestions in the initial ideas-gathering survey. Three hundred and eighty-eight (63%) identified themselves as healthcare professionals; 304 (49%) as patients with experience of surgery or anaesthesia, and 299 (48%) as carers or friends of patients who had  undergone surgery or anaesthesia  Eleven partner organisations (three patient organisations, eight  anaesthetic specialist societies) submitted a further 56 suggestions, yielding 1476 proposed research questions in total. | None. | The wide range of stakeholders involved in each stage of the process means that these 10 topics can  be considered to reflect the mutual research interests of clinicians, patients and carers.  The PSP has also identified  a ‘longlist’ of research topics, most of which, although not selected among the top ten priorities, also  reflect shared research concerns among healthcare professionals  and service users alike. |
| Boote, Jonathan, Wendy Baird, and Anthea Sutton. "Public Involvement in the Systematic Review Process in Health and Social Care: A Narrative Review of Case Examples." Health Policy 102.2 (2011): 105-16. Web. 9 May 2017. | *Design:*  Case study/series  *Setting:*  UK  *Technique:*  Focus Group/ Group Meeting | Six papers reported public involvement in the development of a clinical trial, while one reported public involvement in the development of a mixed methods study.  Method of involvement included focus group meetings and feedback on trial encouraged through questionnaire; Consultation meetings including questionnaires; focus groups; consultation meetings with members of support group; and semi-structured interviews | Tensions between different stakeholder groups; level of understanding of members of the public and health research methods; time and cost; representativeness; and language and jargon. | Interviews carried out by people of same nationally; training of intervieweers; accuracy of interview schedules checked using back translation; educate and confirm understanding of research such as clinical trials; testing understanding of consent; testing effect of consumer involvement in design; ; independent consultants facilitating focus groups; all stakeholders in research involved at very outsiet; collating data by independent researchers; negotiation between stakeholders requires facilitation; allow sufficient time for negotiation and feedback |
| Boote, Jonathan, Wendy Baird, and Claire Beecroft. "Public Involvement at the Design Stage of Primary Health Research: A Narrative Review of Case Examples." Health Policy 95.1 (2011): 10-23. | *Design:*  Case study/series  *Setting:*  UK  *Technique:*  Focus Group/ Group Meeting | The public was found to contribute to systematic reviews by: refining the scope of the review; suggesting and locating relevant literature; appraising the literature; interpreting the review findings; writing up the review. Numerous tensions, facilitating strategies and recommendations  were identified. | Tight timescale imposed by commissioning organization can limit extent of public involvement in review process  Expressed anxiety over complexity of process; Emotional impact of group members being asked to represent an organization  Power/authority differential; time needed to facilitate/build trust | Develop quality standards; payment support and training to maximize inovlvement or public; public should be adequately debriefed before and at review meetings; Develop membership criteria; Funding for public involvement built into the review's grant ; application; use of online Delphi process to reach conensus rapidly and inexpensively; offer glossary of terms to advisory group; Briefing papers sent out before each meeting;  Utilizing a multistep participatory decision  making process with CAB and SC members helped to ensure  that CTSI research and community engagement strategies  are congruent with community priorities.  The structured and  systematic approaches of both techniques ensured that the  diverse perspectives and organizational agendas of each member  were a part of the group’s frame of reference.  Participatory  decision making is a cornerstone of CBPR and key to effective  community–university partnerships. The process described in  this paper can serve as a model for other CTSAs and community–  academic partnerships interested in identifying health  and research priorities to foster shared research collaborations |
| Bragge, P., L. Piccenna, J. W. Middleton, S. Williams, G. Creasey, S. Dunlop, D. Brown, and R. L. Gruen. "Developing a Spinal Cord Injury Research Strategy Using a Structured Process of Evidence Review and Stakeholder Dialogue. Part I: Rapid Review of SCI Prioritisation Literature." Spinal Cord 53.10 (2015): 714. | *Design:*  Literature review  *Setting:*  Australia/ New Zealand  *Technique:*  Global Evidence Mapping (GEM) | N/A | None. | The reviews inform specific research topics and highlight other important research considerations, most notably those pertaining to SCI patients’ perspectives on quality of life, which may be of use in determining meaningful research outcome measures. T  he views of other SCI research stakeholders such as researchers, clinicians, policymakers, funders and carers would help shape a bigger picture of SCI research priorities, ultimately optimising research outputs and translation into clinical practice and health policy change. |
| Brett, Jo, Sophie Staniszewska, Carole Mockford, Sandra Herron‐Marx, John Hughes, Colin Tysall, and Rashida Suleman. "Mapping the Impact of Patient and Public Involvement on Health and Social Care Research: A Systematic Review." Health Expectations 17.5 (2014): 637-50. | *Design:*  Literature review  *Setting:*  UK  *Technique:* N/A | The positive impacts identified enhanced the quality and appropriateness of research. Impacts were reported for all stages of research, including the development of user-focused research objectives, development of user-relevant research questions, development of user-friendly information, questionnaires and interview schedules, more appropriate recruitment strategies for studies, consumer-focused interpretation  of data and enhanced implementation and dissemination of study results. Some challenging impacts were also identified. | Much of the evidence base concerning impact remains weak and needs significant enhancement in the next decade.  There is also evidence of researchers’ tokenistic attitude towards PPI  Time, cost. | Incorporating user views into the research agenda may lead to divergence from scientific methods and cause ethical dilemmas during the protocol design; It is the researcher’s role to ensure  any study is of high quality scientifically  Explain and justify the research design and negotiate changes that make the study more  acceptable to service users without compromising its robustness or validity |
| Brocklehurst, Paul R., Laura Mackay, Joanna Goldthorpe, and Iain A. Pretty. "Older People and Oral Health: Setting a Patient‐centred Research Agenda." Gerodontology 32.3 (2015): 222-28 | *Design:*  Non-experimental  *Setting:* UK  *Technique:*  JLA-PSP | 1. Users of Service; 2. Carers - personal carers or relatives or spouses over the age of 6; 3. Third Sector - those representing third sector organizations such as 'older people's charities and care home staff' and 4. 'Specialists' - those with a specialist knowledge in one or more areas related to the topic e.g., Dental Public Health | None. | The wide range of stakeholders involved in each stage of the process means that these 10 topics can  be considered to reflect the mutual research interests of clinicians, patients and carers. |
| Broerse, Jacqueline E. W., Marjolein B. M. Zweekhorst, Annemiek J. M. L. Van Rensen, and Monique J. M. De Haan. "Involving Burn Survivors in Agenda Setting on Burn Research: An Added Value?" Burns 36.2 (2010): 217-31. | *Design:*  Non-experimental  *Setting:*  Scandinavia  *Technique:*  Dialogue Model | The project followed the methodology of the Dialogue Model for patient participation in research agenda setting. This model is grounded in the so-called responsive research methodology. In a responsive approach the issues of stakeholders are the starting point for a dialogue about the improvement of a certain practice. Stakeholders are people or organizations whose issues are at stake. Their involvement  is based on the premise that each stakeholder has a specific perspective on the issue and that a dialogue among and between the stakeholder groups will result in a better informed, more sophisticated decision on the improvement of practice. The different stakeholders are actively involved in the process of design, data collection, analysis and dissemination. The responsive methodology was originally developed in  the field of program evaluation and then translated to the field of health research | More time should have been taken for truly joint product. This implies that it is better not to include the final priority setting step in the same meeting (making the meeting longer is no option given the fact that it was already quite tiring particularly for many burn survivors). | Investigate to what extent bias has occurred in the priority lists due to the exclusion of some groups in the process –children, adolescents, hospitalized burn survivors, nurses and anaesthesiologists. It could very well be that they have a perspective that is different from the groups whose voices were included. |
| Brooks, Fiona. "Nursing and Public Participation in Health: An Ethnographic Study of a Patient Council." International Journal of Nursing Studies 45.1 (2008): 3-13. | *Design:*  Non/experimental  *Setting:*  UK  *Technique:*  Survey and key informant interviews | The aim is to explore the relationship of the nursing profession to public participation as enacted through a UKbased patient and public council, located in an acute hospital. The council was developed by nursing staff and aimed to achieve service user participation in strategic level health care decision-making. The views and experiences of participants and the applicability of the ‘nurse–patient partnership’ construct to public participation are considered. The study employed integrative ethnography, involving multiple field methods: non-participant observation of council meetings, i.e. fourteen 3 h meetings (n ¼ 42 h); in-depth interviews with councillors (n ¼ 17) and with key hospital staff (n ¼ 18). A documentary review and mapping of the actions of the council was undertaken. | None | Partnership in public participation requires a shift by nurses’ towards acceptance of members of the public functioning as informed, critical and powerful agents in health care decision-making. Equipping nurses with the skills to communicate with patient representatives in a position of interest |
| Chalmers, Iain, Adib Essali, Emtithal Rezk, and Sally Crowe. "Is Academia Meeting the Needs of Non-academic Users of the Results of Research?" The Lancet 380 (2012): S43. Web. 9 May 2017. | *Design:*  Non-experimental  *Setting:*  USA  *Technique:*  JLA-PSP | Overview/analysis of JLA implications on patient representativeness | Invitation for non-academic users of research to identify research priorities seems to lead to research questions that differ importantly from the focus of much of the current health research. The public ends up providing the resources to support academia’s involvement in research. | Researchers need to consider their responsibilities to take account of the needs of public and other users of research and ask themselves what they are doing to ensure that they are meeting these needs.  Non-academic users of research need to engage with the research community to encourage research that addresses their needs. |
| Cheraghi‐Sohi, Sudeh, Peter Bower, Anne Kennedy, Andrew Morden, Anne Rogers, Jane Richardson, Tom Sanders, Fiona Stevenson, and Bie Nio Ong. "Patient Priorities in Osteoarthritis and Comorbid Conditions: A Secondary Analysis of Qualitative Data." Arthritis Care & Research 65.6 (2013): 920-27. | *Design:*  Qualitative interviews  *Setting:*  USA  *Technique:*  Focus Group | Three groups of patients emerged from the analysis.  The 2 smaller groups had stable priorities (where OA was or was not prioritized) and illustrated the importance of factors, such as personal social context and the specific nature of the comorbid conditions.  The third and largest group reported priorities that shifted over time. | Shifting appeared to be influenced by the participants’ perceptions of control and/or interactions with clinical professionals, and could have important consequences for self-management behavior | The various factors underlying patients’ priorities among their conditions, and the fluctuating nature of these priorities, highlight the importance of regular assessments during clinician–patient consultations to allow better communication and treatment planning, and ultimately optimize patient outcomes |
| Chung, Bowen, Arleen F. Brown, Gerardo Moreno, Pattie Cuen, Visith Uy, Sitaram Vangala, Douglas S. Bell, A. Eugene Washington, Keith C. Norris, and Carol M. Mangione. "Implementing Community Engagement as a Mission at the David Geffen School of Medicine at the University of California, Los Angeles." Journal of Health Care for the Poor and Underserved 27.1 (2016): 8. | *Design:*  Non-experimental  *Setting:*  Australia/New Zealand  *Technique:*  Survey & key informant interviews | Key survey findings provided support for the inclusion of community engagement as a core mission at DGSOM and UCLA Health.  Nearly 10% of all invited to take the survey participated in a community- engaged project in the prior year supporting the initial strategic planning stakeholder perspectives that individuals have generally initiated community- engaged projects despite limited institutional support, coordination, or visibility. | None | This proportion of participation in community– engaged work was higher than expected. Over one- quarter of respondents with an Academic appointment and one- third from the UCLA Health indicated interest in learning about best practices for community- engaged research. This high level of interest reinforced the strategic plans’ goal to create a Community Engagement Institute to provide infrastructure and support for existing community engagement activities and opportunities to build capacity through training and education |
| Clavisi, Ornella, Peter Bragge, Emma Tavender, Tari Turner, and Russell L. Gruen. "Effective Stakeholder Participation in Setting Research Priorities Using a Global Evidence Mapping Approach." Journal of Clinical Epidemiology 66.5 (2013): 496-502.e2 | *Design:*  Non-experimental  *Setting:* Australia/New Zeland  *Technique:* | Successfully prioritized 26 research questions using multi staged GEM approach | Limitations of having participants almost exclusively from Australia; does not support international research needs | None |
| Conklin, Annalijn, Zoë Morris, and Ellen Nolte. "What Is the Evidence Base for Public Involvement in Health‐care Policy?: Results of a Systematic Scoping Review." Health Expectations 18.2 (2015): 153-65 | *Design:*  Literature review  *Setting:*  UK  *Technique:*  Focus Group/ Group Meeting | Round tables, town hall meetings, open houses + population-specific events and issue specific groups  Citizen panels, survey, telephone or face to face survey, group meetings, Community Health Councils, local patient groups | Although several studies aimed to assess the influence of public involvement on decisions, policies or practice using ‘change’ as an indicator of effect, few were designed to be comparative or had sufficiently long observation periods to adequately measure such impact on policy and practice in the longer term.  The concept of public involvement that was under study and the indicators used to evaluate and determine any resultant effect/impact were poorly specified and inconsistent. | Continuing absence of a consensus on the definition of public involvement, and the variation in purpose of and approaches to involvement, either of which are often not made explicit |
| Costa-Font, Joan, Joan Rovira Forns, and Azusa Sato. "Participatory Health System Priority Setting: Evidence from a Budget Experiment." Social Science & Medicine (1982) 146 (2015): 182-90. Web. 9 May 2017. | *Design:*  Non-experimental  *Setting:*  UK  *Technique:*  Focus Group/Group meeting | This paper explores the use of a participatory budget experiment (with 88 participants clustered in social groups) to model public health care reform, drawing from a set of realistic scenarios for potential health care users. We measure preferences by employing a contingent ranking alongside a budget allocation exercise (termed ‘willingness to assign’) before and after program cost information is revealed | Cost-estimation bias  Underestimation of the amount of funding required to reimburse highly valued programs that extend health care converge and increase equity of the health system.  Fiscal illusion by individuals that participated in the experiment.  Collective  decision making lies in dealing with individual's reluctance to participate which in turn over-represents the preferences of certain population groups. | How to include a preference for public savings or investment outside health care which influences the health system |
| Cox, Anna, Anne Arber, Ann Gallagher, Mairead Mackenzie, and Emma Ream. Oncology Nursing Forum 44.2 (2017): 192 | *Design:*  Non-experimental  *Setting:*  UK  *Technique:*  Delphi | Consensus building with highest agreement was reached within and across groups on the need for research relating to prevention, screening, early diagnosis, and psychological care across the cancer trajectory. Little consensus was reached regarding symptoms and side effects. | Oncology nurses and patients do not necessarily prioritize the same research areas.. | Prevention, screening, and early diagnosis are of the highest priority for future research among oncology nurses and patients. |
| D'Andreamatteo, Carla, Karen M. Davison, and Pat Vanderkooy. "Defining Research Priorities for Nutrition and Mental Health: Insights from Dietetics Practice." Canadian Journal of Dietetic Practice and Research : A Publication of Dietitians of Canada = Revue Canadienne De La Pratique Et De La Recherche En Diététique : Une Publication Des Diététistes Du Canada 77.1 (2016): 55. | *Design:*  Non-experimental  *Setting:*  Canada  *Technique:*  Survey and key informant interviews | Respondents prioritized different nutrition and mental health research questions by rating them using a Likert-type scale of importance followed by selecting the 1 research question that was the most important within each of the 3 categories: (i) improve the health of people living with mental health conditions (5 questions); (ii) improve community nutrition and mental health programs and services (3 questions); and (iii) promote mental health and/or prevent or delay the onset of mental health conditions (7 questions).  The second prioritizing step included a review of all of the research questions and selecting the top 3 research questions the participants believed would have the greatest impact on nutrition and mental health in the community. | As an inductive exploratory pilot study with a population drawn from a convenience sample, it is not known if the findings are generalizable to all dietitians.  In addition, not all stakeholder groups could be analyzed in depth due to limited subset samples. | Amid these limitations, however, this foundational work has identified dietitians’ priorities for nutrition and mental health research which can help advance practice. |
| Domecq, Juan Pablo, Gabriela Prutsky, Tarig Elraiyah, Zhen Wang, Mohammed Nabhan, Nathan Shippee, Juan Pablo Brito, Kasey Boehmer, Rim Hasan, Belal Firwana, Patricia Erwin, David Eton, Jeff Sloan, Victor Montori, Noor Asi, Abd Moain Abu Dabrh, and Mohammad Hassan Murad. "Patient Engagement in Research: A Systematic Review." BMC Health Services Research 14.1 (2014): 89. | *Design:*  Literature Review  *Setting:*  USA  *Technique:*  Focus Groups/ Group Meeting/ Workshop | We found no comparative analytic studies to provide evidence supporting a particular method to identify or select patients for engagement in research; Involved focus group, individual interview, survey and deliberation | None | None |
| Elberse, J. E., J. F. Caron-Flinterman, and J. E. W. Broerse. "Patient-expert Partnerships in Research: How to Stimulate Inclusion of Patient Perspectives." Health Expectations 14.3 (2011): 225-39. | *Design:*  Non-experimental  *Setting:*  Scandinavia  *Technique:* Dialogue Model | The Dialogue Model is grounded in participatory and interactive approaches and has been adjusted on the basis of pilot work. It has six phases: exploration; consultation; prioritization; integration; programming; and implementation.  The data are clustered using a framework that divides exclusion mechanisms in three categories: circumstances [caused by setting of the dialogue - location, who is invited, place, time and duration], behaviour [Behaviour of participants or facilitator, less speaking time, attention or respect] and verbal communication [use of jargon, sidelining of issues, subjective or not feasible to include all perspectives] | In this case patients and patient representatives – or their perspectives are not taken up in the decision-making process, because of actions taken by members of other stakeholder groups or the process facilitator. Exclusion can be intended or unintended and can be induced by various mechanisms. | None |
| Elwyn, Glyn, Sally Crowe, Mark Fenton, Lester Firkins, Jenny Versnel, Samantha Walker, Ivor Cook, Stephen Holgate, Bernard Higgins, and Colin Gelder. "Identifying and Prioritizing Uncertainties: Patient and Clinician Engagement in the Identification of Research Questions." Journal of Evaluation in Clinical Practice 16.3 (2010): 627. | *Design:*  Non-experiential  *Setting:*  UK  *Technique:*  JLA-PSP | A four-step procedure: (1) establish a collaborative Working Partnership; (2) identify and collect treatment uncertainties by using a patient survey and analysing existing systematic reviews, clinical guidelines and query-answering services; (3) categorize uncertainties; and (4) convene a workshop using a nominal group process to establish a ranked prioritization of treatment uncertainties in asthma. The estimated costs of supporting the WP process were £29 000, excluding approximately 6 months of work at DUETs. | None | Smaller task groups can help complete the identification of treatment uncertainties, develop priority setting methods and identify and prioritize uncertainties |
| Etchegary, Holly, Lisa Bishop, Catherine Street, Kris Aubrey-Bassler, Dale Humphries, Lidewij Eva Vat, and Brendan Barrett. "Engaging Patients in Health Research: Identifying Research Priorities through Community Town Halls." BMC Health Services Research 17 (2017): n. pag. | *Design:*  Non-experimental  *Setting:*  Canada  *Technique:*  Focus groups/Group Meeting | Town hall meetings - Eight town halls were held with members of the general public in rural and urban settings across the province. Sessions were a hybrid information-consultation event, with key questions about health research priorities and outcomes guiding the discussion.  The team was comprised of two clinicians, a pharmacist, and health re searchers from a variety of areas (e.g., genetics, public health, primary care). | Communication assistance was not offered for town halls, which could have precluded participation  from some members of the public. No translation services were thought to be required as the population in this jurisdiction is largely homogenous (white, English-speaking, and middle class), and none was required | Public research priorities and suggestions for improving the provision of healthcare provide valuable  information to guide Support Units planning and priority-setting processes. A range of research areas were raised  as priorities for patients that are likely comparable to other healthcare systems. These create a number of health  research questions that would be in line with public priorities. Findings also provide lessons learned for others and  add to the evidence base on patient engagement methods. |
| Flaman, Laura M., Candace I. J. Nykiforuk, Ronald C. Plotnikoff, and Kim Raine. "Exploring Facilitators and Barriers to Individual and Organizational Level Capacity Building: Outcomes of Participation in a Community Priority Setting Workshop." Global Health Promotion 17.2 (2010): 34-43 | *Design:*  Non-experimental  *Setting:*  Canada  *Technique:*  groups/Group Meeting | This study consisted of three separate phases. The first involved analysis of ANGEL-CD Workshop participants’ ranking of priority strategies derived from work sheets used at the ANGEL-CDWorkshops.  Overall, participants identified strategies that sought to change the physical environment highly (total scores ranged from 38.1% to 61.9%). In phase two, individuals’ capacity to adopt chronic disease  prevention initiatives within their organization was assessed through administration of a self-report survey. Analysis indicated that participants had high individual leadership, moderate individual ‘will’ and  infrastructure, and low organizational ‘will’ and infrastructure. | First, the constant comparative technique used for analysis is susceptible to bias.  Study processes relied on the participants’ recall of information from the ten months leading up to the interview.  Bias may have also resulted from the nature of the sample. Nine of 11 participants worked for nonprofit organizations, while the two others worked for government. | Confirm that participant organizations are supportive of the initial goals of priority setting meetings to ensure emergent priorities can be addressed; decentralize resources to meet increasing demands  to allow for efficient and effective health promotion; address larger community issues prior to, or in conjunction with, dealing with smaller community priorities; ensure that CDP strategies are focused within the community of interest and account for community context; and bring together groups and individuals who already share common goals and views with respect to solutions for problems. Recognition of these elements will allow practitioners, organizations, governments/funders, and communities to focus on seeking ways to improve capacity for chronic disease prevention.  Future studies should examine variation between organizational types to identify similarities and differences in the capacity building process. |
| Forsythe, Laura P., Lauren E. Ellis, Lauren Edmundson, Raj Sabharwal, Alison Rein, Kristen Konopka, and Lori Frank. "Patient and Stakeholder Engagement in the PCORI Pilot Projects: Description and Lessons Learned." Journal of General Internal Medicine 31.1 (2016;2015;): 13-21 | *Design:*  Literature review  *Setting:*  USA  *Technique:*  n/a | Surveying stakeholders to identify research priorities | n/a | Surveying stakeholders and planning meetings with significant lead time  Hold meetings less frequently or with fewer stakeholders  Provide maximum compensation Seeking out support from existing resources at their institution Expand their stakeholder recruitment by networking within the group of stakeholders already engaged  Researcher training on cultural sensitivity and community engagement |
| Ingram, J. R., R. Abbott, M. Ghazavi, A. B. Alexandroff, M. Mcphee, T. Burton, and T. Clarke. "The Hidradenitis Suppurativa Priority Setting Partnership." British Journal of Dermatology 171.6 (2014): 1422-427. | *Design:*  Non-experimental  *Setting:*  UK  *Technique:*  JLA-PSP | Survey 1 took place from March to April 2013 and 1495 potential uncertainties were submitted, 57% by patients with HS and carers, 24% by dermatologists, 12% by GPs, 4% by dermatology specialist nurses and 3% by other HCPs including surgeons and psychodermatologist Survey 2 occurred from October to November 2013 and was returned by 371 participants, 50% of whom were HS patients and carers  The steering committee decided that, in the context that HS is relatively under-researched, there were many important unanswered non treatment questions that mattered to both patients and their clinicians. This view was confirmed by four non-treatment uncertainties featuring in the top 10 priorities | None | Strong patient and carer involvement, in particular support from the HS Trust, was very important in balancing the perspectives of clinicians and consumers in the process, as advocated by the JLA ethos  Of note, there was a nearly equal contribution of both groups to each of the surveys. In addition, each final workshop subgroup contained at least three patients or carers, to ensure that clinicians did not dominate |
| Jonas, Daniel E., Alyssa J. Mansfield, Pam Curtis, John H. Gilmore, Lea C. Watson, Shannon Brode, Sonia Tyutyulkova, Karen Crotty, Meera Viswanathan, Elizabeth Tant, Cathy Gordon, Samantha Slaughter-Mason, and Brian Shetiman. "Identifying Priorities for Patient-Centered Outcomes Research for Serious Mental Illness." Psychiatric Services 63.11 (2012): 1125-130 | *Design:*  Non-experimental  *Setting:*  USA  *Technique:*  JLA-PSP | To generate topics, three meetings were held; the first and third meetings were held by conference call with Web-hosted presentations, and the second was an all-day, in-person meeting at AHRQ. A professional  facilitator was used during all While the JLA provides guidelines and a framework for the methodology of PSPs, a pragmatic approach is taken so the process can be adapted to meet the differing requirements of  PSP’s.  A thematic analysis approach was developed to manage, refine and develop research questions from the very large amount of collected uncertainties. Moreover, each question was checked against an extensive and complex evidence base of system meetings At the first meeting, participants provided feedback in the areas related to research on serious mental illness that they identified as having greatest interest.  We invited participants to continue the discussion and submit research ideas by e-mail,phone, or an online forum hosted by AHRQ. The suggestions by participants  were grouped into three main themes: patient-centered care— improving outcomes that matter to patients; conceptual frameworks for research; and reducing disparities for subpopulations. | Resource constraints limited the number and scope of participants. Similarly, although the most productive part of the process was the in-person meeting, resources limited face-to-face  Many participants were not initially familiar with the EHC program and PCOR and required the context provided by the optional orientation sessions about the EHC program and PCOR and about how PCOR  might benefit them. This critical step allowed participants, especially those who were not clinicians or researchers, to participate fully. At the end of the process, most participants indicated a basic understanding of PCOR and the EHC program and a willingness to continue participating in similar processes. Consequently, investigators and individual stakeholders are working together to advance priority topics for consideration in the EHC program. | Engagement by a broad group of stakeholders in a transparent process resulted in the identification of priority areas for PCOR. Stakeholders clearly indicated a need to fundamentally change how research on serious mental illness is conducted and a critical need for the development of methodology and infrastructure. Most current PCOR has been focused on relatively short-term outcomes, but real world, long-term studies providing guidance for treatment over the lifetime of a serious mental illness are needed. |
| Kelly, Sarah, Louise Lafortune, Nicola Hart, Katherine Cowan, Mark Fenton, Carol Brayne, and Dementia Priority Setting Partnership. "Dementia Priority Setting Partnership with the James Lind Alliance: Using Patient and Public Involvement and the Evidence Base to Inform the Research Agenda." Age and Ageing 44.6 (2015): 985-93 | *Design:*  Non-experimental  *Setting:*  UK  *Technique:* JLA-PS | Thematic analysis was used to identify themes from the large amount of questions collected from which research questions were developed using 985 Dementia priority setting partnership with the James Lind Alliance PICO framework  Each question was checked against an extensive evidence base of high-quality systematic reviews to verify whether they were true uncertainties.  One thousand five hundred and sixty-three questionnaires were received, from people with dementia, carers/relatives, and health and care professionals; 85 uncertainties were identified from other sources. Questions were refined and formatted iteratively into 146 unique uncertainties. An interim prioritization process involving diverse organizations identified the top 25 ranked questions. At a final face-to-face prioritization workshop, 18 people representing the above constituencies arrived by consensus at the top 10 priority questions. The impact of patient and public involvement on the priorities is discussed. | None | They highlight a need for more research into care for people with dementia and carers, and a need for high quality effectiveness trials in all aspects of dementia research.  The long (146 questions) and top 10 lists of dementia research priorities provide a focus for researchers, funders and commissioners. |
| Khodyakov, Dmitry, Susan E. Stockdale, Nina Smith, Marika Booth, Lisa Altman, and Lisa V. Rubenstein. "Patient Engagement in the Process of Planning and Designing Outpatient Care Improvements at the Veterans Administration Health‐care System: Findings from an Online Expert Panel." Health Expectations 20.1 (2017): 130-45. | *Design:*  Qualitative interviews  *Setting:*  USA  *Technique:*  Focus group/Group meetings | Delphi survey (Expert Lens) to distinguish between 8 scenarios of patient roles as consultant, implementation advisor, equal stakeholder, lead stakeholder at either the local-level or regional-level care planning and design decision making Criteria included feasibility, patient input, physician/staff acceptance, patient-centeredness, health-care quality, overall desirably | Structural barriers to public participation have been highlighted and include uncertainty over the practicalities of promoting patient involvement,  the precise role the public should play, poorly resourced integration into systems for service improvement and professional attitudes to patient involvement.  A commonly cited concern is that patients knowledge and awareness focus predominantly on their personal elements of care, with few patients having the awareness and ⁄ or motivation to understand the broader perspectives required for involvement in strategic health service planning. | Health-care systems may need to provide multiple engagement opportunities so that patients could choose the one that best fits their interests, skills and preferences. |
| Kielmann, Tara, Guro Huby, Alison Powell, Aziz Sheikh, David Price, Sian Williams, and Hilary Pinnock. "From Awareness to Involvement? A Qualitative Study of Respiratory Patients’ Awareness of Health Service Change." Health Expectations 14.3 (2011): 321-33. | *Design:*  Qualitative interviews  *Setting:*  UK  *Technique:*  Focus groups/ group meetings | Participants were not only aware of trends in health service provision (e.g. emergence of new professional roles, shift from secondary to primary care) but interpreted changes in the light of  local and national events. Despite this awareness, none of the patients was formally involved in service development, though some contributed to local voluntary groups.  Professionals generally welcomed the need for patients views to be heard. | Practical obstacles most often cited (e.g., structural barriers) | Prioritize patient voice and mechanisms to support patient experience |
| Knight, Simon R., Leanne Metcalfe, Katriona O’Donoghue, Simon T. Ball, Angela Beale, William Beale, Rachel Hilton, Keith Hodkinson, Graham W. Lipkin, Fiona Loud, Lorna P. Marson, and Peter J. Morris. "Defining Priorities for Future Research: Results of the UK Kidney Transplant Priority Setting Partnership." Plos One 11.10 (2016): E0162136. | *Design:*  Non-experimental  *Setting:*  *UK*  *Technique:*  JLA-PSP | The PSP methodology is as outlined by the James Lind Alliance. An initial survey collected unanswered research questions from patients, carers and clinicians. Duplicate and out-of scope topics were excluded and the existing literature searched to identify topics answered by current evidence. An interim prioritization survey asked patients and professionals to score the importance of the remaining questions to create a ranked long-list. These were considered at a final consensus workshop using a modified nominal group technique to agree a final top ten. | The current process may under-represent certain groups affected by transplantation. In particular, very few responses were received from children and adolescents despite partnership  with children’s renal charities and the British Association  A further limitation of the current process is the relatively small number of participants in the final workshop. A group size of around 25 participants was determined to provide optimal  interaction based upon experience from previous JLA PSPs, and within the budget limitations of the project. | List of priorities invaluable to researchers and funders for direct future activity |
| Layton, Alison, E. Anne Eady, Maggie Peat, Heather Whitehouse, Nick Levell, Matthew Ridd, Fiona Cowdell, Mahenda Patel, Stephen Andrews, Christine Oxnard, Mark Fenton, and Lester Firkins. "Identifying Acne Treatment Uncertainties via a James Lind Alliance Priority Setting Partnership." BMJ Open 5.7 (2015): E008085 | *Design:*  Non-experimental  *Setting:*  UK  *Technique:*  JLA-PSP | Treatment uncertainties were collected via separate online harvesting surveys, embedded within the PSP website, for patients and professionals. A wide variety of approaches were used to promote the surveys to stakeholder groups with a particular emphasis on teenagers and young adults. Survey submissions were collated using keywords and verified as uncertainties by appraising existing evidence. The 30 most popular themes were ranked via weighted scores from an online vote. At a priority setting workshop, patients and professionals discussed the 18 highest-scoring questions from the vote, and reached consensus on the top 10. | Large volume of unsorted questions; need method to sort and share large volumes of submissions | To ensure all views were captured, much effort went into collecting responses from a wide spectrum of people with acne and different types of care professionals; despite this, males with acne were under-represented.  Grouping into broad themes was the only way of generating manageable numbers to take to the prioritisation stages without overwhelming participants.  Saturation was reached in that no new uncertainties were contained within the final submissions to the harvesting survey. |
| Lophatananon, Artitaya, Sandy Tyndale‐Biscoe, Emma Malcolm, Helen J. Rippon, Kate Holmes, Lester A. Firkins, Mark Fenton, Sally Crowe, Sarah Stewart‐Brown, Vincent J. Gnanapragasam, and Kenneth Ross Muir. "The James Lind Alliance Approach to Priority Setting for Prostate Cancer Research: An Integrative Methodology Based on Patient and Clinician Participation." BJU International 108.7 (2011): 1040-043. | *Design:*  Non-experimental  *Setting:*  UK  *Technique:*  JLA-PSP | JLA Method Stage 1. Gathering the uncertainties; Stage 2: Consultation process to refine uncertainties; Stage 3: Verification of true uncertainties; Stage 4: First ranking exercise; and Stage : Priority setting workshop | The limitation here again lies with the limited numbers present in the group discussion stage, which reflected both lack of availability and perhaps some apathy on behalf of some clinicians .  Uneven number of patients and clinicians could have produced biased results for the two parties. | Future initiatives could be improved by achieving a greater number and better balance of numbers between |
| Madden, Mary, and Richard Morley. "Exploring the Challenge of Health Research Priority Setting in Partnership: Reflections on the Methodology Used by the James Lind Alliance Pressure Ulcer Priority Setting Partnership." Research Involvement and Engagement 2 (2016): n. pag. | *Design:*  Non-experimental  *Setting:*  UK  *Technique:*  JLA-PSP | JLA Method Stage 1. Gathering the uncertainties; Stage 2: Consultation process to refine uncertainties; Stage 3: Verification of true uncertainties; Stage 4: First ranking exercise; and Stage : Priority setting workshop  Submissions were categorised as follows: Intervention/non-intervention Originator (source) Submission group (patient/service user, carer, health professional, mixed)  Broad intervention taxonomy derived from the Cochrane Wounds group: organisation of care; pressure and shear reduction and relief; local wound treatment; managing patients with limited mobility; risk assessment; nutrition; local skin care; surgery; other. Detailed intervention taxonomy derived from the Cochrane Wounds group e.g. local wound treatment (dressings, topical treatments etc.) | A significant proportion of people with pressure ulcers find it difficult to participate in activities outside their homes due to immobility, frailty and co-morbidities.  There were (at that time) no pressure ulcer or wounds specific existing patient/service user groups.  Online working may only be suitable for small numbers of patients and service users.  care staff for pressure ulcer prevention interventions. The professional community involved therefore spans NHS and Social Care providers including private health and social care.  The JLA does not engage with industry in its processes because commercial interests may not align with scientific or public interests. This may challenge professionals used to working with industry as a major sponsor of health care research and education. | The introduction of open-ended questioning also proved to be a valuable addition to the value-weighting survey method, with added scope for understanding why participants allocated their funding as they did |
| Medlow, S., and P. Patterson. "Determining Research Priorities for Adolescent and Young Adult Cancer in Australia: AYA Research Priorites." European Journal of Cancer Care 24.4 (2015): 590-99 | *Design:* Non-experimental  *Setting:* Australia/New Zealand  *Technique:*  Survey, Key informant interviews | In order to assess stakeholders’ research priorities, we adapted a ‘value-weighting’ survey approach that has been used previously to determine research priorities for  Australian patients with blood cancers  The underlying technique is to ‘. . . provide experts with a scenario similar to that faced by decision makers – what proportion of funding should be  directed towards each of the available research options? Conducting value weighting is a two-step process (1) reviewing the literature and using expert opinion to determine a set of potential topics of interest such as areas of priority research and priority research populations; and (2) using the topics determined in step 1 as the basis of a survey in which health professionals and consumers provide rankings of research sub-topics to indicate where investment funds should be spent. | While great care was taken in selecting research topics for the present value-weighting study, we recognize that a pre-determined list cannot be exhaustive and that other topics may have been omitted  inadvertently | None |
| Miller, Caroline L., Kathy Mott, Michael Cousins, Stephanie Miller, Anne Johnson, Tony Lawson, and Steve Wesselingh. "Integrating Consumer Engagement in Health and Medical Research – an Australian Framework." Health Research Policy and Systems 15.1 (2017): n. pag | *Design:*  Non-experimental  *Setting:*  Australia/New Zealand  *Technique:*  Survey, Key informant interviews | Process with the aim of developing an evidence-based, best-practice (consumer partnership), practical framework for consumer and community engagement in research process for the Institute, with a remit for an entire jurisdiction – the State of South Australia A mixed-method, iterative process was undertaken to identify available evidence, gather experiences and views of stakeholders (namely consumers, researchers, and academic experts in consumer engagement) and engage those stakeholders in the design of a consumer and community engagement framework for health and medical research. To further embed a consumer perspective in the development of the framework, a partnership was established with the local peak body for health consumers | Comprehensive examples of consumer participation in health and medical research are limited.  There are few documented studies of what techniques are effective. | This evidence-driven framework, developed in collaboration with consumers, is being integrated in a health and medical research institute with diverse programs of research. This framework is offered as a contribution to the evidence base around meaningful consumer engagement and as a template for other research institutions to utilize. |
| Mitton, Craig, Neale Smith, Stuart Peacock, Brian Evoy, and Julia Abelson. "Public Participation in Health Care Priority Setting: A Scoping Review." Health Policy 91.3 (2009): 219-28. | *Design:*  Literature review  *Setting:*  Canada  *Technique:*  Focus group/group meeting/workshop | Key literature review findings suggest that there has been a steady growth in number of empirical case studies of public engagement over recent years in health, environment, urban planning sectors  All levels of government report efforts to engage public  Public engagement is most common at visioning or goal setting  Purposive recruitment is most common, with some use of random self-selection  A range of methods are used; cost is seldom reported however well-structured can range from thousands to million plus range and engagement exercises are rarely formally evaluated | n/a | n/a |
| Nierse, C. J., and T. A. Abma. "Developing Voice and Empowerment: The First Step towards a Broad Consultation in Research Agenda Setting." Journal of Intellectual Disability Research 55.4 (2011): 411-21. | *Design:*  Non-experimental  *Setting:*  Scandinavia  *Technique:*  Survey and key informant interviews | Seven persons with ID and six parents were interviewed individually. Subsequently, 10 focus groups were organised with people with ID and four focus groups with parents. Also, a questionnaire was sent to parents | n/a | The process of developing intimate voice and political voice can be regarded as a concretisation of enclave deliberation among disempowered groups. These steps are necessary to initiate a process towards establishing a broad consultation between different stakeholders about research on ID |
| Northway, Ruth, Karen Hurley, Chris O'Connor, Helen Thomas, Joyce Howarth, Emma Langley, and Sue Bale. "Deciding What to Research: An Overview of a Participatory Workshop." British Journal of Learning Disabilities 42.4 (2014): 323-27. | *Design:*  Non-experimental  *Setting:*  UK  *Technique:*  Focus group/ group meeting/ workshop | The afternoon started with two brief presentations: the first related to what we mean by research and the second to the local strategy for the development of services for people with learning disabilities. The latter was included as it was felt important to note what people had felt to be important in terms of service development as this might influence their thinking about research  Having set the scene participants were asked to work in small (mixed) groups and to note down the areas where they felt research should take place. This process led to many lively discussions and debate. Clearly some people felt that issues of personal importance to them because of their life experience should be examined while others took a broader view. | Time commitment  Planning - Particularly focused on client/community needs -- eg disability | The importance of careful planning was recognised early in this process, and a small planning group was formed. This comprised one woman with learning disabilities, members of Health Board staff and members of University staff. |
| Ottersen, Trygve, Reidun Førde, Meetali Kakad, Alice Kjellevold, Hans Olav Melberg, Atle Moen, Ånen Ringard, and Ole Frithjof Norheim. "A New Proposal for Priority Setting in Norway: Open and Fair." Health Policy 120.3 (2016): 246-51 | *Design:*  Non-experimental  *Setting:*  Scandinavia  *Technique:*  Focus group/group meeting/ workshop | The framework comprises four general principles. Priority setting should: pursue the goal of “the greatest number of healthy life years for all, fairly distributed”; be based on clear criteria; be open, systematic, and involve user participation; and be supported by a coherent set of effective instruments.. | n/a | The three criteria can be used either to directly rank competing interventions or together with pre defined thresholds. Cost-effectiveness thresholds have been introduced informally in Norway, but never examined and approved by Parliament. The Committee found that such thresholds could help facilitate priority-setting based ont he three proposed criteria. In particular, thresholds were seen as useful in situations where the decision-maker has little information about the interventions competing for the same resource |
| Pandya-Wood, Raksha, Duncan S. Barron, and Jim Elliott. "A Framework for Public Involvement at the Design Stage of NHS Health and Social Care Research: Time to Develop Ethically Conscious Standards." Research Involvement and Engagement 3 (2017): n. pag. | *Design:*  Non-experimental  *Setting:*  Scandinavia  *Technique:*  n/a |  | Advocate for sufficient time for public involvement  Avoid tokenism  Register with research design stage public involvement work early with NHS Research Department  Communicate clearly from outset | Build in realistic timelines; Make public contributions viable; Use guidance from funding bodies; Include as much information as possible, in plain language  Consider group facilitation skills; Inform people early on about their involvement; Respect and value all forms of difference in individuals; Avoid research terminology; Don’t combine qualitative research methods and public involvement activities |
| Patten, San, Craig Mitton, and Cam Donaldson. "Using Participatory Action Research to Build a Priority Setting Process in a Canadian Regional Health Authority." Social Science & Medicine 63.5 (2006): 1121- | *Design:*  Qualitative Interviews  *Setting:*  Canada  *Technique:*  Participatory action research (PAR) | Combined qualitative data collection methods of document review, participant observation, in-depth interviews and focus groups. Throughout the PAR process, the health economists participated in the  priority setting exercise not only as researchers, but also as consultants. Participant observation notes were taken during all priority setting meetings and training presentations, as well as during the  focus groups and interviews. | The PAR process was not linear and did not follow discrete stages as the researchers initially envisioned.  Action, research and change interventions interacted throughout the PAR project as dynamic and overlapping processes.  This fluidity in the process of PAR makes it difficult to actually measure the impact that the health economists exerted upon the change  process. | The unpredictable nature of action research necessitates a flexible rather than a rigid research design. |
| Perros, Petros, Colin M. Dayan, A. Jane Dickinson, Daniel G. Ezra, Janis L. Hickey, Christoph Hintschisch, George Kahaly, John H. Lazarus, Marian Ludgate, Beate Bartès, Caroline J. Macewen, Anna L. Mitchell, Dan Morris, Nicole O'Connor, Simon H. Pearce, Geoffrey E. Rose, Mario Salvi, Wilmar M. Wiersinga, Alyson Williamson, Thyroid Eye Disease Amsterdam Declaration Implementation Group UK, European Group On Graves’ Orbitopathy, and On Behalf Of the Thyroid Eye Disease Amsterdam Declaration Implementation Group UK (TEAMeD) and European Group on Graves' Orbitopathy (EUGOGO). "Future Research in Graves' Orbitopathy: From Priority Setting to Trial Design Through Patient and Public Involvement." Thyroid 25.11 (2015): 1181-184. | *Design:*  Non-experimental  *Setting:*  UK  *Technique:*  JLA-PSP | Day 1 was aimed at patients, the public, experts, and professionals. It included an introductory session about GO for patients and the public, patient testimonies about their experience with the disease, and a breakout session into three working groups.  Day 2 consisted of a scientific meeting aimed at professionals and six invited expert patients. It included reviews of the pathogenesis and clinical management of GO, highlighting recent advances in these areas. It was followed by a breakout session of three working groups for professionals, experts, and expert patients, allocated to translating the selected research questions formulated in breakout session 1, to study design outlines.  The objectives of breakout session 1 were to discuss six questions per group previously set by the Sight Loss and Vision Priority Setting Partnership (and select a maximum of two questions per group based on scientific merit and relevance to unmet patient needs.  Groups in breakout session 2 consisted of a chair, co-chair, expert panelists (including two expert patients per group) and professionals. They were asked to discuss and translate the PICO questions of breakout session 1 into study designs. |  |  |
| Piil, Karin, and Mary Jarden. "Patient Involvement in Research Priorities (PIRE): A Study Protocol." BMJ Open 6.5 (2016): E010615. | *Design:*  Qualitative interviews  *Setting:*  Scandinavia  *Technique:*  Focus group/ group meeting | Focus Group Interviews - 8-10 participants; representative from a relevant patient organization, patients, relatives and specialists create the most comfortable environment  Use of moderate, assistant moderator and semistructured interview guide inspired by the JLA method | n/a | n/a |
| Pittens, C. A. C. M, J. E. Elberse, M. Visse, T. A. Abma, and J. E. W. Broerse. "Research Agendas Involving Patients: Factors That Facilitate or Impede Translation of Patients' Perspectives in Programming and Implementation." Science and Public Policy 41.6 (2014): 809-2 | *Design:*  Non-experimental  *Setting:*  Scandinavia  *Technique:* Dialogue Model | Model operationalizes consultation of and collaboration between various stakeholders and is grounded in notion that involvement is an interactive process between stakeholders  Key 6 steps include: Initiation and preparation; Consultation; Prioritization; Integration; Programming; and Implementation | n/a | n/a |
| Pollock A, St George B, Fenton M, et al. Development of a new model to engage patients and clinicians in setting research priorities. J Health Serv Res Policy 2013;19:12–18 | *Design:*  Non-experimental  *Setting:*  UK  *Technique:*  Survey and key informant interview | A model to facilitate involvement through targeted engagement and assisted involvement (FREE TEA model). We implemented both standard surveys and the FREE TEA model to gather research priorities (treatment uncertainties) from people affected by stroke living in Scotland. We explored and configured the number of treatment uncertainties elicited from different groups by the two approaches. | Focused on underrepresented populations with complex health needs; cannot use standard surveys which may result in poor or underrepresentative involvement of patients, thereby favouring views of professionals | n/a |
| Pratt, Bridget, Maria Merritt, and Adnan A. Hyder. "Towards Deep Inclusion for Equity-oriented Health Research Priority-setting: A Working Model." Social Science & Medicine 151 (2016): 215-24. | *Design:*  Non-experimental  *Setting:*  USA  *Technique:*  Deep Inclusion | A. Planning Phase [1.Set aims of priority setting process 2. Identify mechanism for priority setting (e.g., series of plenaries, small group work); 3. Set ground rules for process (e.g., how chair is selected, rules for speaking); 4. Determine who is to participate in the priority setting process; 5. Develop strategies for promoting qualitative equality during priority setting; 6. Determine mode of non-elite participation for priority setting  B. Identify reserach questions and criteria phase [7. Identify health research topics/questions; 8. Identify ranking criteria; 9. Identify weights for ranking criteria]  C. Selecting priorities phase [10. Applying ranking criteria and weights to health research topics/questions; 11. Determine a final set of priority health reserach topics/questions] | Inherent challenges to implementing include inherent tension between including a diverse set of participants and keeping numbers low enough to facilitate deliberation; identifying who the relevant non-elites are; establishing pre-conditions for research beneficiaries' involvement, limited resources and lack of political will to support inclusive deliberative process;  Need time and resources  Political assumptions as 'value-laden and political' process in which various stakeholders' agenda will typically involve considerations other than achieving inclusion.  Identifying the specific factors that drive qualitative inequality in given country contexts and how to combat those factors to achieve deep levels of non-elite participation. | Consider equity focus to ensure voices of patients are heard in priority setting |
| Regier, Dean A., Colene Bentley, Craig Mitton, Stirling Bryan, Michael M. Burgess, Ellen Chesney, Andy Coldman, Jennifer Gibson, Jeffrey Hoch, Syed Rahman, Mona Sabharwal, Carol Sawka, Victoria Schuckel, and Stuart J. Peacock. "Public Engagement in Priority-setting: Results from a Pan-Canadian Survey of Decision-makers in Cancer Control." Social Science & Medicine 122 (2014): 130-39 | *Design:*  Non-experimental  *Setting:*  Canada  *Technique:*  n/a | Pan-Canadian survey of decision-makers in cancer control to investigate the types of evidence, especially evidence supplied by the public, that are utilized in health care priority-setting.  The results indicated that public engagement was infrequently utilized compared to clinical effectiveness evidence or cost evidence. General positive agreement between normative attitudes towards the use of evidence and the frequency of evidence utilization was observed, but absence of correlative agreement was found for the types of evidence that are supplied by the general public and for cost-effectiveness inputs.  Regression analyses suggested that  public engagement was unevenly utilized between jurisdictions and that educational background and barriers to implementing public input may decrease the odds of using public engagement as evidence.  We recommend that institutions establish a link between committee members' normative attitudes for using public engagement and its real-world utilization. | n/a | Committees establish a strong link between members' normative views on using public values and the real-world utilization of such evidence.  Public engagement exercises emphasizing deliberative democracy may address several barriers to using  public input, particularly with respect to decision-makers’ trust in  the public's ability to provide input for priority-setting processes. |
| Restall, Gayle J., Tara N. Carnochan, Kerstin Stieber Roger, Theresa M. Sullivan, Emily J. Etcheverry, and Pumulo Roddy. "Collaborative Priority Setting for Human Immunodeficiency Virus Rehabilitation Research: A Case Report." Canadian Journal of Occupational Therapy. Revue Canadienne D'ergothérapie 83.1 (2016): 7. | *Design:*  Non-experimental  *Setting:*  Canada  *Technique:*  Focus group/ group meeting/ workshop | World Café/Dotmocracy 2 day forum  The two-day forum was designed to include people living with HIV, researchers, service providers, and policy makers. Due to the anticipated diversity of our participants, we wanted the most inclusive and equalizing approach to foster conversations to identify priority research topics. The World Cafe´ methodology facilitates small-group discussion in an intimate setting inviting spoken words as well as written words and pictures. As in any dialogue, dominant and articulate voices could prevail, leaving some voices out. Therefore, we also used Dotmocracy, a voting procedure, as an approach to final decision making.  Participants perceived the World Cafe´ format as encouraging a safe environment for respectful dialogue. | Although the hosts tried to avoid the use of complex language and jargon, there were times when people used language that may have been difficult for others in the group to understand. The Dotmocracy process, on the other hand, allowed each participant to cast their votes anonymously and nonverbally, giving equal weight to each participant in the final determination of priorities. | To facilitate transparency of how decisions about priorities were established, the World Cafe´ generated ideas and topics, which were then grouped into categories by the whole group, an activity that is common in the initial stages of qualitative data analysis  The researchers’ presentation of the topics at the beginning of the 2nd day was consistent with member checking  Dotmocracy provided a means to order the topics according to votes contributed by everyone.  To facilitate equalization of the voices of more articulate participants with those who were less verbally expressive, participants were encouraged to contribute in ways that felt most comfortable for them: speaking, drawing, or doodling. This process allowed those who were less verbally expressive to have other ways of communicating their idea were involved in multiple transparent stages of priority setting |
| Rideout, Catlin, Rosa Gil, Ruth Browne, Claudia Calhoon, Mariano Rey, Marc Gourevitch, and Chau Trinh-Shevrin. "Using the Delphi and Snow Card Techniques to Build Consensus Among Diverse Community and Academic Stakeholders." Progress in Community Health Partnerships: Research, Education, and Action 7.3 (2013): 331-39. | *Design:*  Non-experimental  *Setting:*  USA  *Technique:*  Delphi | The Delphi technique is a methodology used to generate consensus from diverse perspectives and organizational agendas through a multi-method, iterative approach to collecting data. A series of on-line surveys was conducted with CAB members to identify health and research priorities from the community perspective. Subsequently, CAB and SC members were brought together and the snow card approach was utilized to narrow to two priority areas for shared research collaborations. snow card approach was utilized to narrow to two priority areas for shared research collaborations. | n/a | The Delphi approach fostered ownership and engagement with community partners because it was an iterative process that required stakeholders’ input into decision making. The snow card technique allowed for organizing of a large number of discrete ideas. Results have helped to inform the overall CTSI research agenda by defining action steps, and setting an organizing framework to tackle two health disparity areas. The process helped ensure that NYUHHC CTSI research and community engagement strategies are congruent with community priorities. |
| Sibbald, Shannon L., Peter A. Singer, Ross Upshur, and Douglas K. Martin. "Priority Setting: What Constitutes Success? A Conceptual Framework for Successful Priority Setting." BMC Health Services Research 9.1 (2009): 43 | *Design:*  Literature  *Setting:*  Canada  *Technique:*  n/a | Conceptual framework elements to prioritize in health research includes stakeholder engagement as part of the process (which includes public and patients) as well as outcomes - i.e., stakeholder understanding  Process – 1. Stakeholder engagement 2. Explicit process; 3. Information management; 4. Consideration fo context and values; and 5. Revision of appeals mechanisms  Outcome: 6. Stakeholder understanding; 7. Shifted priorities/reallocation of resources; 8. Improved decision making quality; 9. Stakeholder acceptance and satisfaction/ 10. Positive externalities. | n/a | n/a |
| Sofolahan-Oladeinde, Yewande, Robin P. Newhouse, Danielle C. Lavallee, Jennifer C. Huang, and C. Daniel Mullins. "Early Assessment of the 10-step Patient Engagement Framework for Patient-centred Outcomes Research Studies: The First Three Steps." Family Practice (2017): n. pag. | *Design:*  Qualitative  *Setting:*  USA  *Technique:*  n/a | Perceptions of patient engagement in health research  Our overall findings illustrate that patient engagement creates an alignment between patients’ and researchers’ priorities yet remains challenging to integrate into current research culture. The impact is  that researchers are able to better prioritize important patient outcomes and design the research study using language and messaging that resonates with patients. In addition, our findings indicate that  having a representative sample of patients to engage who are able to provide valuable input on different aspects of the disease area was challenging for some | Our findings highlight factors, which influence patient engagement, such as inadequate institutional support and tight deadlines of funding agencies. Creating infrastructure within research institutions publicized and easily accessible to researchers, especially new and untenured faculty, facilitates patient engagement | Examples of such infrastructure could include providing small institutional/ departmental pilot funds for pre-engagement, establishing a work group, organizing seminars, establishing collaborative interdisciplinary  partnerships between new and experienced PCOR researchers to serve as mentoring opportunities and to give access to already developed patient networks and partnerships |
| Truitt, A, Monsell, S, Avins, A, Nerenz D, Lawrence, S, Comstock, B … Lavalee D. Prioritizng research topics: a comparison of crowdsourcing and patient registry. Quality of Life Research, 2017. doi:10.1007/s11136-017-1566-9 | *Design:*  Quasi-experimental  *Setting:*  USA  *Technique:*  Survey and key informant interview | The two approaches resulted in similar research priorities by frequency.  Both provided open-ended responses that were useful, in that they illuminate additional and nuanced research topics.  Overall, both approaches suggest a preference towards topics related to diagnosis and treatment over other topics. | n/a | Using a patient registry and crowdsourcing are both feasible recruitment approaches for engagement. Researchers should consider their approach, community, and resources when choosing their recruitment approach, as each approach has its own strengths and weaknesses. These approaches are likely most appropriate to supplement or to complement in-person and ongoing engagement strategies |
| Van Merode, Tiny, Silvia Bours, Ben Van Steenkiste, Theo Sijbers, Gerard Van Der Hoek, Cor Vos, Gerard M. J. Bos, and Trudy Van Der Weijden. "Describing Patients’ Needs in the Context of Research Priorities in Patients with Multiple Myeloma or Waldenstrom's Disease: A Truly Patient-driven Study." Zeitschrift Fuer Evidenz, Fortbildung Und Qualitaet Im Gesundheitswesen 112 (2016): 11-18. | *Design:*  Non-experimental  *Setting:*  UK  *Technique:*  Dialogue Model | Establish balanced project group and agreed on study protocol; actual data collection including individual interviews and focus groups with purposeful sample of patients and questionnaire that was sent to all members of patient organizations and a dialogue meeting with patients to prioritize final issues |  | Ensure patient perceptive is central  Questionnaire built with themes and topics that came directly from patients point of view and patties were able to add items they missed in questionnaire |
| Van Middendorp, J. J., H. C. Allison, S. Ahuja, D. Bracher, C. Dyson, J. Fairbank, A. Gall, A. Glover, L. Gray, W. El Masri, A. Uttridge, and K. Cowan. "Top Ten Research Priorities for Spinal Cord Injury: The Methodology and Results of a British Priority Setting Partnership." Spinal Cord 54.5 (2016): 341. | *Design:*  Non-experimental  *Setting:*  UK  *Technique:*  JLA-PSP | This partnership involved the following four key stages: (i) gathering of research questions, (ii) checking of existing research evidence, (iii) interim prioritisation and (iv) a final consensus meeting to reach agreement on the top ten research priorities. Adult individuals with spinal cord dysfunction because of trauma or non-traumatic causes, including transverse myelitis, and individuals with a cauda equina syndrome (henceforth grouped and referred to as SCI) were invited to participate in this priority setting partnership. | n/a | Comprehensive rigorous and inclusive participation process with individuals, caregivers and health professionals informs the best scope for future activities of funders and researchers |
| Walton, Nancy A., Douglas K. Martin, Elizabeth H. Peter, Dorothy M. Pringle, and Peter A. Singer. "Priority Setting and Cardiac Surgery: A Qualitative Case Study." Health Policy 80.3 (2007): 444-58 | *Design:*  Survey and key informant interviews  *Setting:*  Canada  *Technique:*  Case study/series | Examine the process of priority setting processes at three University of Toronto affiliated cardiac surgery centres. In data analysis, the conditions of "accountability for reasonableness" (relevance, publicity, appeals and enforcement) were used as an analytic lens.  While decisions may appear to be based strictly upon clinical criteria (e.g. coronary anatomy); non-clinical criteria also have an impact upon decision-making (e.g. patients' lifestyle choices, type of surgical practice and departmental constraints on resource use). Participants stated that these factors influence their decision-making and can result in unfair and inconsistent decisions | n/a | Four conditions to promote accountability for reasonableness include 1. Relevance condition – rationales for priority setting decisions must rest on reasons and principles that fair-minded persons agree are relevant in meeting the diverse needs of population within the context of resource constraints; 2. Publicity condition – limit setting decisions and their rationales must be publicly accessible; 3. Appeals condition – mechanism for challenge and disputing decision so f limit setting as well as opportunity to revisit decision in light of further evidence; and 4 – enforcement condition. Voluntary or public regulation to ensure first three conditions are met |
| Watson, Verity, Andrew Carnon, Mandy Ryan, and Derek Cox. "Involving the Public in Priority Setting: A Case Study Using Discrete Choice Experiments." Journal of Public Health (Oxford, England) 34.2 (2012): 253-60 | *Design:*  Non-experimental  *Setting:*  UK  *Technique:*  Discrete Choice Experiment | The weighted benefit score is a composite measure that both scores a project against pre-defined criteria and assigns each criterion a weight. Visual analogue and Likert-type scales ranging from 1 to 10 or 1 to 100 have been used to score health-care services. Visual analogue and Likert-type scales are problematic because many services obtain similar scores. The weights assigned to each criterion are value judgements about the relative importance of a criterion to the overall service benefit. Some studies have not assigned weights6 implicitly assuming all criteria are equally important, others have used existing evidence from the literature and/or local expert opinion to assign weights. | n/a | A wide perspective by considering priority setting at the local NHS organization level and by defining benefits in terms of a broad range of attributes including health outcomes and benefits such as access to services and national priorities. This allows us to compare bids across many clinical and non-clinical areas. |
