# Supplementary material for: Patient and public engagement in priority setting: A systematic rapid review of the literature
Source: PLoS One. 2018 Mar 2;13(3):e0193579. doi: 10.1371/journal.pone.0193579 (PMC5834195; doi:10.1371/journal.pone.0193579)
Supplement: S1 Fig — (DOC) [file pone.0193579.s005.doc]

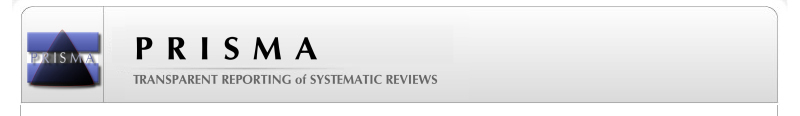
**PRISMA 2009 Flow Diagram**

**Screening**

**Included**

**Eligibility**

**Identification**

Records identified through database searching
(n = 536 )

Additional records identified through other sources
(n = 0)

Records after duplicates removed
(n = 7 )

Records screened
(n = 529 )

Records excluded
(n = 451 )

Full-text articles assessed for eligibility
(n = 78 )

Full-text articles excluded, with reasons
(n = 8 )

Studies included
(n =70 )
